# Chemoenzymatic synthesis of polypeptides in neat 1,1,1,2-tetrafluoroethane solvent†

**DOI:** 10.1039/c8ra06657d

**Published:** 2018-10-22

**Authors:** Isabel S. Aguirre-Díaz, Carmina Montiel, Ismael Bustos-Jaimes, Yaocihuatl Medina-Gonzalez, Alberto Tecante, Miquel Gimeno

**Affiliations:** Facultad de Química, Depto. de Alimentos y Biotecnología, Universidad Nacional Autónoma de México (UNAM) CDMX Mexico mgimeno@unam.mx; Facultad de Medicina, Depto. de Bioquímica, Universidad Nacional Autónoma de México (UNAM) CDMX Mexico; INPT, UPS, Laboratoire de Genie Chimique UMR CNRS 5503, Universite de Toulouse 4, Allee Emile Monso F-31030 Toulouse France

## Abstract

Chemoenzymatic polypeptide synthesis offers several advantages over chemical or other biological routes, however, the use of aqueous-based media suffers from reverse hydrolysis reactions that challenge peptide chain propagation. Herein, the protease from subtilisin Carlsberg biocatalyzed the synthesis of poly-l-PheOEt, poly-l-LeuOEt, and the copolymers poly-l-PheOEt-*co*-l-LeuOEt from their amino acid ethyl ester substrates in a neat liquid 1,1,1,2-tetrafluoroethane solvent. The products, achieved in acceptable yields (*ca.* 50%), were fully characterized showing relatively high molar mass (*ca.* 20 000 Da for poly-l-PheOEt). This non-toxic low-boiling hydrofluorocarbon enhances enzymatic peptide propagation by limiting hydrolysis owing to its hydrophobic and relatively polar characteristics that sustain the protease activity and solubilize substrates and products. Computational molecular dynamic calculations were used to assess the l-PheOEt/l-LeuOEt-solvent and polypeptide-solvent interactions in this system. Additionally, the homopolypeptides displayed higher crystallinity than the copolypeptides with random incorporation of amino acid ethyl esters, notwithstanding the significantly highest specificity for Phe in this system. Interestingly, secondary structure characterization of the products by FTIR and circular dichroism suggests a non-common peptide folding.

## Abstract 2

Chemoenzymatic polypeptide synthesis offers several advantages over chemical or other biological routes, however, the use of aqueous-based media suffers from reverse hydrolysis reactions that challenge peptide chain propagation.

## Graphical abstract

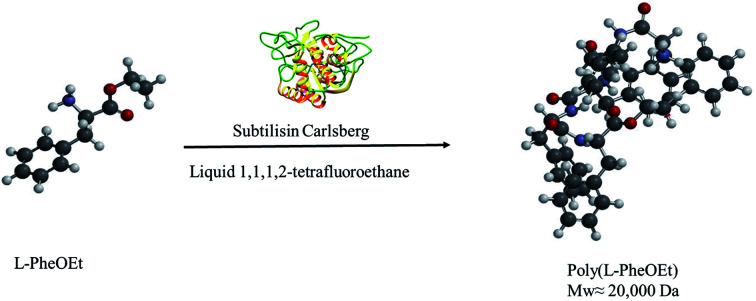

## Introduction

Synthetic polypeptides are interesting materials as they can mimic the characteristics of proteins.^1–3^ In this regard, the polypeptide primary and secondary structures are crucial for a wide range of applications as advanced and smart materials.^4^ Therefore, research of polypeptides increases more and more from controlled drug delivery, the targeting of specific biological responses to self-assembly studies and hydrogel formation towards bioactive materials, among others.^5,6^ For their synthesis, the Merrifield solid phase, to attain sequence-controlled short peptide structures, is currently the most used,^7^ although there are some limitations due to excessive costs, low product yield derived from time-consuming steps, and expensive purifications. Another chemical approach is the one-step ring opening polymerization from *N*-carboxy anhydride monomers.^5^ However, the approaches based on the enzymes offers mild and more environmentally friendly conditions with the absence of toxicity and side reactions,^6^ which might result in improved polypeptide characteristics. The protease-mediated syntheses of oligopeptides in pure aqueous media or water mixed with organic solvents, *i.e.* MeOH, DMSO, DMF, proved successful.^8^ Therein, the use of highly hydrophilic media allows the solubility of the amino acid-based substrates, mainly amino acid esters while keeping adequate enzymatic activities. To do so, the subtilisin Carlsberg (SC) protease is mostly employed but also papain, trypsin, and chymotrypsin, among others.^6^ However, despite favorable biocatalytic properties in these media, the chain propagation is challenging owing to the reversible hydrolytic reaction. Recently, the bioengineering of microbial proteases, amidases, and peptiligases has emerged in the sought for efficient peptide synthesis, macrocyclization and segment condensation in water.^9–14^ Other approaches include the stability of these enzymes in neat organic solvents or co-solvent systems, thus improving substrates and products solubilities.^15^ Additionally, it is worth mentioning recent advances on transpeptidation and macrocyclization of peptides by sortase and butelase ligase enzymes, although these require specific segment recognition.^16,17^ In general, these reports render low molar mass products when polypeptide synthesis from amino acid substrates or the use of non-commercial enzymes that increase the production costs.

Generally, the use of neat hydrophobic organic media such as toluene, hexane, or hydrophobic ionic liquids for enzymatic polymer synthesis is restricted to commercial lipases to produce manly polyesters. These biotransformations are enhanced by the low polarity of substrates and products and good lipase enzyme stabilities.^18^ Additionally, these enzyme-mediated polymer synthesis includes non-toxic and green compressed fluids (CF)s solvents with remarkable advantages compared to other media regarding low toxicity and green processes.^19–21^ The hydrophobic and relatively polar compressed 1,1,1,2-tetrafluoroethane in combination with commercial lipases produced the poly-l-lactide and branched polyesters.^20^ This organic CF is non-toxic with no ozone depleting potential (ODP) and approved for biomedical uses under the generic name of norflurane, *i.e.* propellant for metered dose inhalers.^22,23^ This low-boiling hydrofluorocarbon becomes easily liquid under small pressure and solubilizes relatively polar substrates while keeping its hydrophobic and aprotic characteristics to inhibit depolymerization and more importantly, to sustain hydrolase-type activities for biotransformations.^24–26^ This work is first to describe a chemoenzymatic synthesis of polypeptides in this neat fluid (313.15 K, 25 bar). For this purpose, l-leucine and l-phenylalanine ethyl esters were used as model substrates and the consequent products were attained in higher yields and molar masses than previous protease-mediated approaches. Additionally, computational molecular dynamic (MD) studies and the Flory–Huggins parameter (*δ*) and energy of mixing calculations assessed the substrates and products interactions with the solvent for this novel system.

## Results and discussion

### Solubility of monomers in the CFs and SC activity in the liquid 1,1,1,2-tetrafluoroethane

The view-cell experiments corroborated the solubility of monomers in organic CF solvent under the experimental conditions. Noteworthy, the initial experiments conducted in liquid CO_2_ as well as in its supercritical state displayed null solubility of the monomers and the presence of carbamates from degradation of the amino acid esters. Evidence of the latter by ^1^H NMR spectroscopy and the lack of solubility ruled out the use of this inorganic CF in our system (see ESI Data 2† for representative ^1^H NMR spectrum of a produced carbamate).

The residual activity of SC samples after treatment in the organic CF up to 48 h showed no significant differences to that for the untreated enzyme. These results provide further evidence for the sustained SC activity to allow biotransformations in this low-boiling hydrofluorocarbon as reported by Micklefield and co-workers at the UMIST (UK) in transesterification reactions.^26^ generally, the activities of hydrolases in hydrophilic organic solvents miscible with water decrease after prolonged exposure. The change in polarity in the active site arising from water partitioning to the media explains this effect. However, water has low miscibility in this organic CF (1 g L^−1^ at 313.15 K), which precludes for water stripping. Additionally, the hydrogen-bonding capacity associated with fluorine atoms might be relevant in keeping the configuration at the enzyme active site at the operational temperatures, as reported by Yu *et al.*^24^ Therefore, the evidence might suggest that the polarity and hydrophobicity of this organic CF, as well as the hydrogen-bonding capacity of the fluorine moieties, might explain the SC activity in this system.

### SC-mediated polypeptide syntheses and characterization

**Fig. 1:**
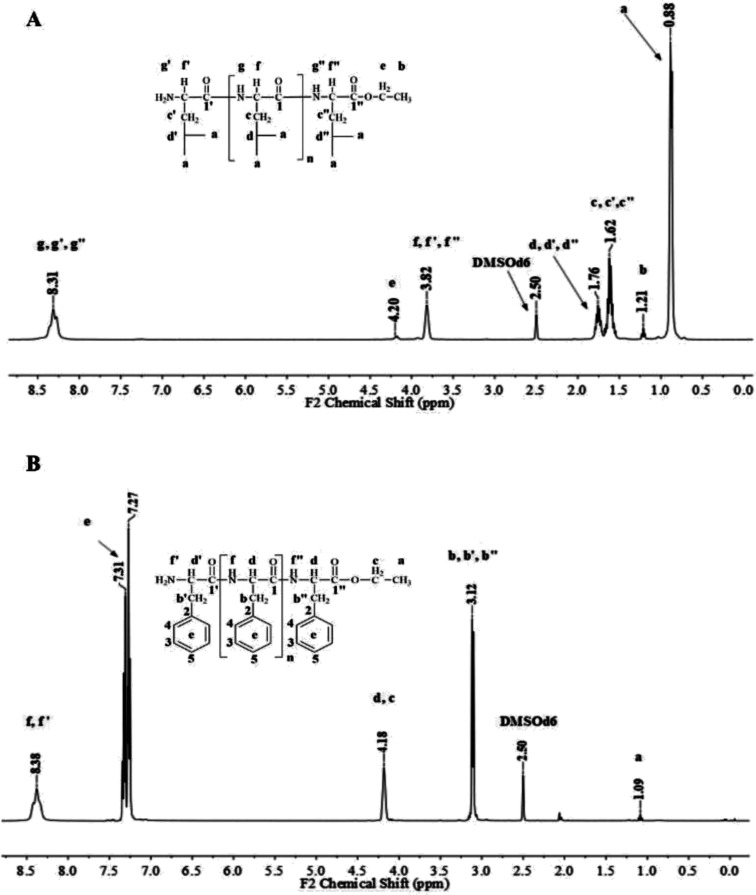
Representative ^1^H NMR spectra with signal assignments for poly(l-LeuOEt) (A); poly(l-PheOEt) (B).

**Fig. 2:**
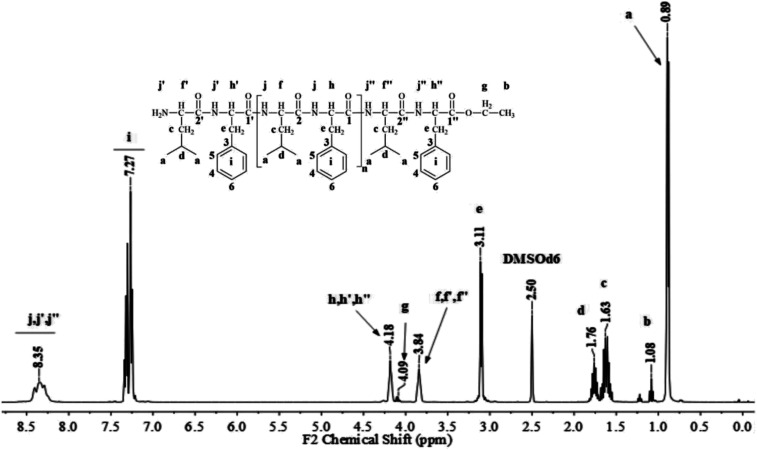
Representative ^1^H NMR spectrum with signal assignments for poly(l-LeuOEt-*co*-l-PheOEt).

Fig. 1 and 2 show the representative ^1^H NMR spectra for the enzymatically synthesized homopolypeptides and co-polypeptides, respectively, with the signal assignments (see ESI Information 3† for the complete 1D and 2D NMR spectra with signal assignments in products).

**Fig. 3:**
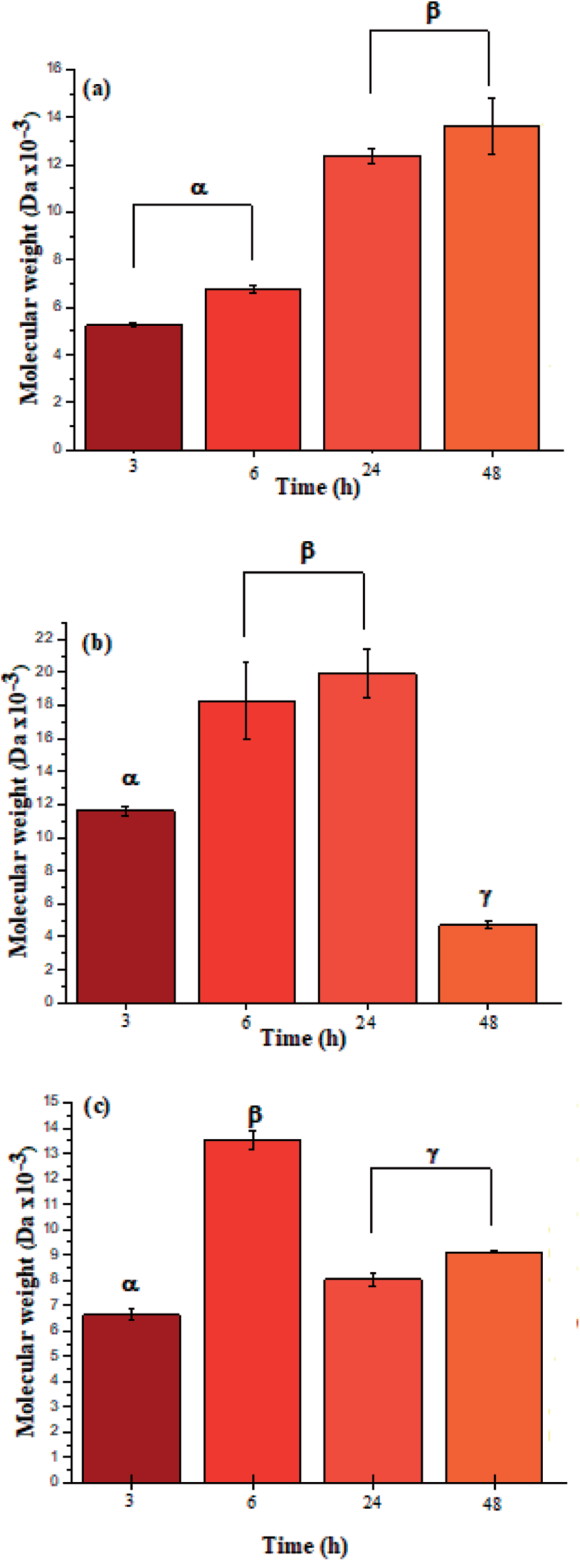
Molar mass distributions calculated by integration of the characteristic ^1^H NMR signals for polypeptides poly(l-LeuOEt) (a); poly(l-PheOEt) (b), and poly(l-LeuOEt-*co*-l-PheOEt) (c).

The ATR-FTIR bands at 1550 cm^−1^ also corroborated the presence of amide bonds (see ESI Information 4† for the ATR-FTIR spectra and band assignments).^4^ Additionally, the UV spectra for l-PheOEt substrates differs from that for poly(l-PheOEt), as shown in the ESI File 5,† and with the typical absorption of peptides at 215 nm, which provides further evidence on this reaction. The relationships between the product yields and molar mass distributions to reaction times are shown in Table 1 and Fig. 3a–c, respectively.

**Table 1:** Polypeptide reaction yields and crystallinity percentages

Entry	Time (h)	Yield (%)	Crystallinity^a^ (%)
Poly(l-LeuOEt)	3	48.19	86.0
Poly(l-LeuOEt)	6	38.04	88.7
Poly(l-LeuOEt)	24	51.26	96.0
Poly(l-LeuOEt)	48	43.79	91.6
Poly(l-PheOEt)	3	51.48	73.1
Poly(l-PheOEt)	6	50.51	87.8
Poly(l-PheOEt)	24	42.91	65.6
Poly(l-PheOEt)	48	42.73	68.7
Poly(l-LeuOEt-l-PheOEt)	3	51.88	55.6
Poly(l-LeuOEt-l-PheOEt)	6	46.48	62.2
Poly(l-LeuOEt-l-PheOEt)	24	49.94	69.1
Poly(l-LeuOEt-l-PheOEt)	48	68.85	69.6

a Data from the integration of crystalline and amorphous areas in the PXRD spectra.

**Fig. 4:**
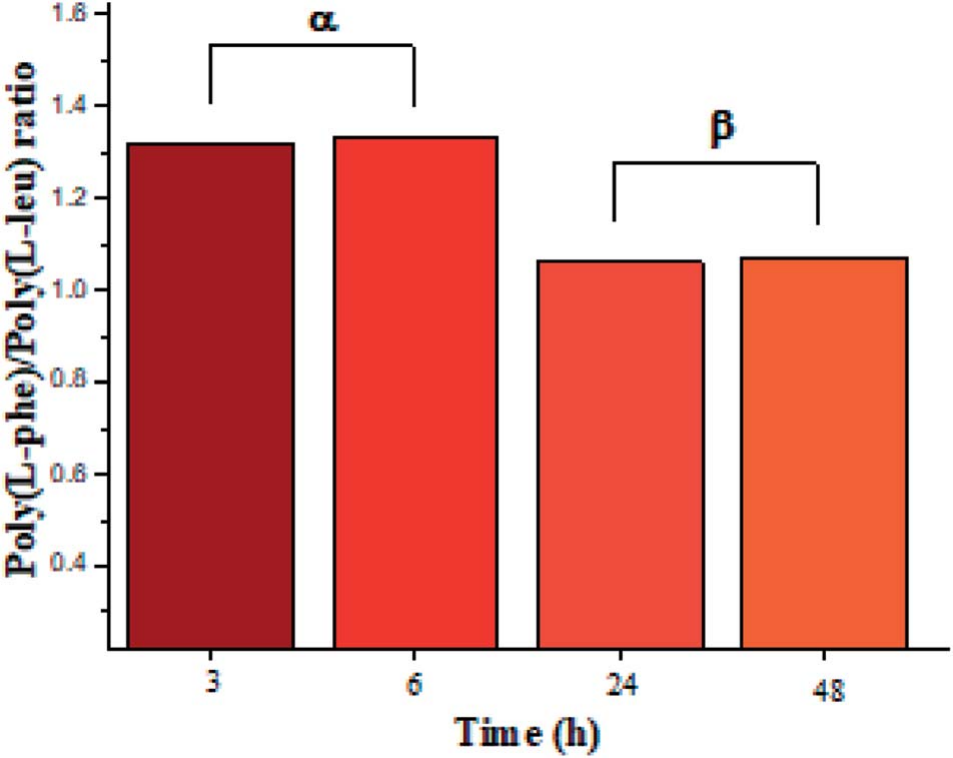
l-PheOEt/l-LeuOEt repeat unit ratio calculated by integration of the characteristic ^1^H NMR signals for each repeat unit.

The recovered products after purification show no variation in yields but in molar masses, which suggests that propagation occurs by the condensation of peptide segments rather than successive incorporation of amino acid ester units. Interestingly, after 24 h, the maximum molar mass of poly(l-PheOEt) (Fig. 3b) decreases significantly, while that for poly(l-LeuOEt) (Fig. 3a) increases until 48 h. The decrease in molar mass but not in yields for the latter as well as in the copolymers (Fig. 3c) evidences that the reaction reached the equilibrium. This condition rules out an earlier termination by precipitation of the growing peptide chains from the media, which was corroborated by the view-cell experiments. Noteworthy, in addition to hydrolysis reaction, the alcoholysis, as transesterification with the released ethanol may occur, producing ethyl esters, which in turn are substrate for the reaction. On the other hand, there is a dramatic molar mass decrease for poly(l-PheOEt) at longer times, which might be ascribed to the alcoholysis or proteolysis, but an opposite behavior for poly(l-LeuOEt) as it increases weight after 24 h. This experimental evidence might be related to the different enzymatic recognition, which is also apparent for poly(l-LeuOEt-*co*-l-PheOEt). In the co-polypeptide syntheses, the maximum molar mass was at 24 h (Fig. 3c), and it tends to decrease with the decrease of l-Phe repeat units (Fig. 4).

In this regard, several authors reported substrate specificity in SC for hydrophobic amino acid residues, particularly for Phe benzene ring, although others showed that it might also depend on the reaction media.^27–29^ In the present study, the enzyme was significantly specific for the most hydrophobic l-Phe units in short reaction time products whereas tends to the equimolar ratio in longer reaction times as shown in Fig. 4. The evidence of this behavior in SC accounts for the prevalence of Phe unit recognition as the co-polypeptide reaction proceeds, which also prompts for random incorporation of repeat units in all the synthesized co-polypeptides. Li *et al.* reported the influence of pH and type of enzyme, *i.e.* SC, on the production of oligopeptides of poly(l-PheOEt) in aqueous-based media. Their strategy was to minimize enzymatic activity losses by addition of co-solvents such as DMSO, DMF, EtOH, MeOH and ACN which also helped to solubilize the as well as products.^30^ The use of this organic CF solvent rules out the control of the reaction pH allowing the solubilities of substrates and products as well as the preservation of the enzymatic activity which explains the enhanced propagation of the polypeptide chains. Noteworthy, control reactions (24 h) without the addition of the enzyme resulted in only 7.05% yield of poly(l-PheOEt) with a molar mass of 661 Da. The same reaction for poly(l-LeuOEt) attained only 6.85% yield of a 520 Da oligomer (see ESI File 6† for a graphical representation of the results in control reactions). Therefore, this demonstrates the contribution of the biocatalyst to the growing peptide chains in this system. Worth to note, the liquid 1,1,1,2-tetrafluoroethane is highly miscible with conventional organic solvents^22^ and other CFs such as compressed CO_2_ (ref. 31) as well as ionic liquids.^32^ Therefore, enzymatic studies in this system might be extended with other co-solvent mixtures.

**Fig. 5:**
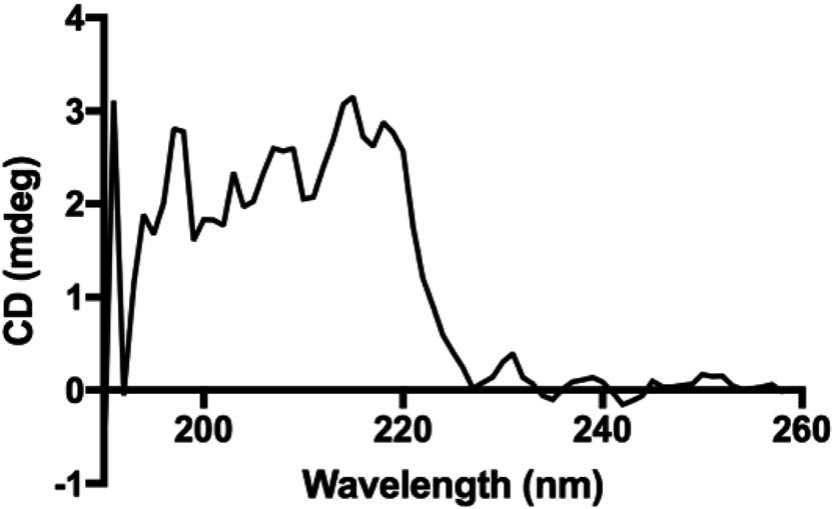
CD spectrum for poly(l-PheOEt) in aqueous solution.

Interestingly, the secondary structure in our polypeptides does not match α-helix or β-sheet folding according to the reported assignments on FTIR spectra in the solid state.^4^ The reports assign amide I bands at 1656 cm^−1^, 1650 cm^−1^ and 1630 cm^−1^ for coils, α-helix, and β-sheets, respectively. For the corresponding amide II bands, they are assigned at 1535 cm^−1^, 1546 cm^−1^ and 1530 cm^−1^ for coils, α-helix, and β-sheets, respectively.^4,33^ However, in our recorded FTIR spectra (ESI File 4†) two bands at 1605 cm^−1^ and 1580 cm^−1^ were always observed for all samples. Additionally, the band at 1730 cm^−1^ in the poly(l-LeuOEt) spectra might be due to terminal units. This signal is probably overlapped by the bands for monosubstituted phenyl in poly(l-PheOEt) and poly(l-LeuOEt-*co*-l-PheOEt) spectra. As described before, to the best of our knowledge, there is no data for secondary structure in early reported enzyme-mediated polypeptides to compare to our data. Additionally, the CD spectrum for poly(l-PheOEt) shown in Fig. 5 agrees with FTIR data as could not be assigned to common protein folding nor that reported for chemically-mediated polypeptides.^4,5^ Nonetheless, the phenyl moieties must hinder water in a secondary structure; properties that might be worth to investigate. Another feature was the high crystallinity of the polypeptides in the PXRD spectra, as shown in Table 1 (see ESI File 7† for PXRD diffraction patterns of the enzymatically synthesized polypeptides).

The percentage of crystallinity does not correlate to the molar mass of the polypeptides. However, there was a decrease as the molar mass decreased for poly(l-PheOEt) at 24 and 48 h reactions, and the opposite occurred for poly(l-LeuOEt) for the same reaction times, concomitantly to its molar mass increase. In addition to this trend, the crystallinity was significantly higher for homopolypeptides as compared to the co-polypeptides, which evidences an increasing disorder among the peptide chains in the case of the latter. Nevertheless, the relation between molar mass and crystallinity with the secondary structure of the enzyme-mediated polypeptides in this organic CF would remain an open question that is worth exploring.

### Computational modeling studies

#### Molecular modeling for density and solubility parameters

**Fig. 6:**
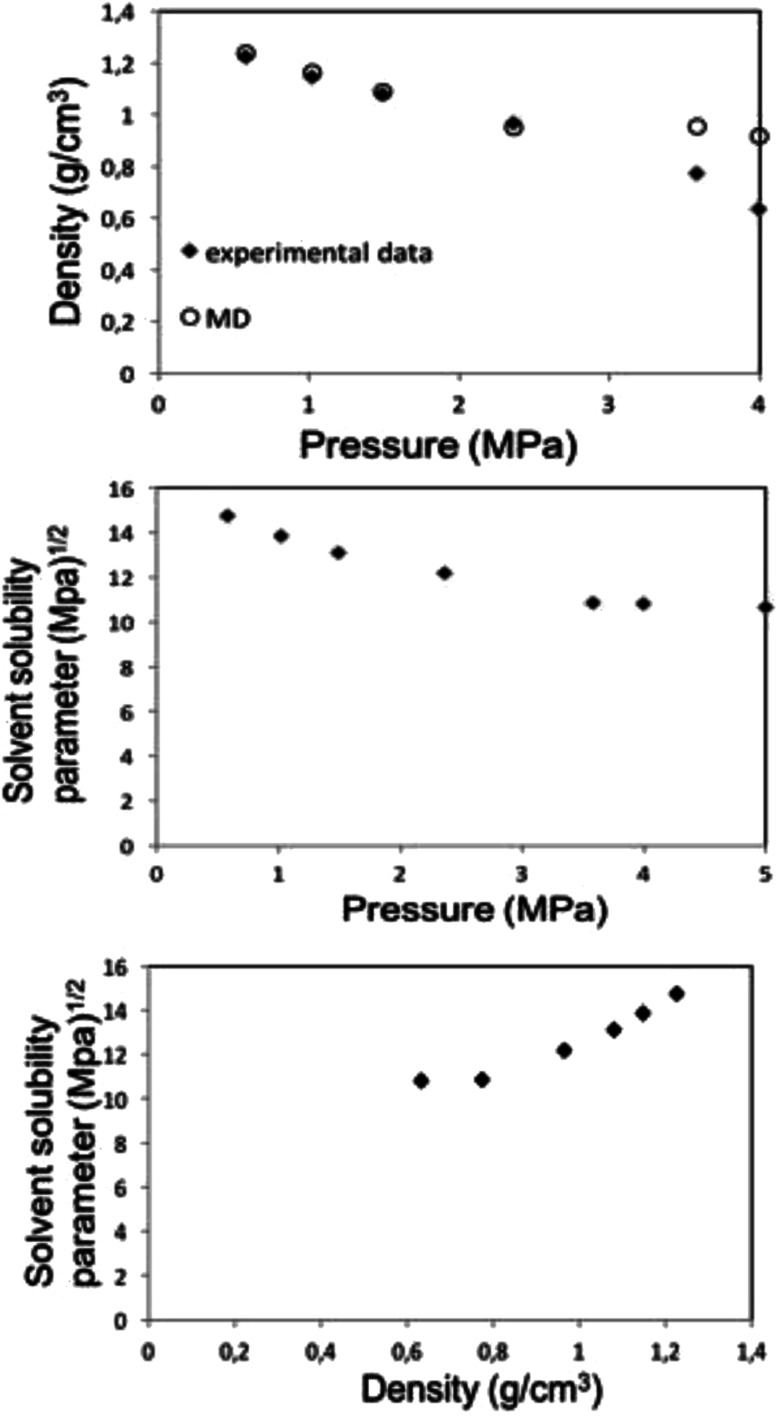
MD calculation for liquid 1,1,1,2-tetrafluoroethane (313.15 K; 25 bar) density (top) and solubility (center) parameters as a function of pressure along the bubble point curve, experimental data taken from Blanke *et al.*^34^ Liquid 1,1,1,2-tetrafluoroethane solubility parameter as a function of density (below).

Fig. 6 shows the MD calculations for the density and solubility parameter of the liquid 1,1,1,2-tetrafluoroethane (313.15 K, 25 bar) together with literature data along the bubble point curve,^34^ as well as the variation of the solubility parameter with density.

**Fig. 7:**
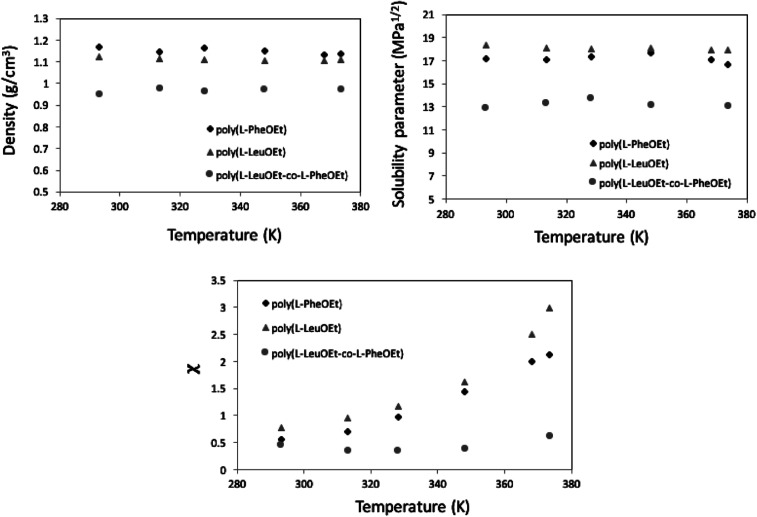
MD calculation as a function of temperature, for density (top) and Hildebrand solubility parameters (center) for the polypeptides. Flory–Huggins parameter for 1,1,1,2-tetrafluoroethane (313.15 K; 25 bar)-polypeptide systems (below).

Along the bubble curve, the pressure increase is related to a temperature increase. Thus, the decrease of the solubility parameter is mainly due to the increase of the temperature which then is in line with the evolution of the Kamlet–Taft parameter (π*), in agreement to the solvatochromic measurements by Abbott and Eardley (1999).^31^ In another related work, Lagalante and co-workers reported an increase in π* with density in this solvent together with an increase in hydrogen-bond acceptor ability parameter (*β*) with density.^35^ In turn, our MD simulations predict an increase in the solubility parameter with density. On the other hand, the Flory–Huggins parameter for the solvent–polymer and solvent–copolymer systems in Fig. 7 display an increase in this parameter, which indicates a decrease in miscibility.

This behavior is more pronounced for the two polymers and decreases as poly(l-PheOEt) > poly(l-LeuOEt) > poly(l-LeuOEt-*co*-l-PheOEt) indicating a better solubility of the copolymer in this organic CF than that for the two polymers individually.

#### Energy of mixing

**Fig. 8:**
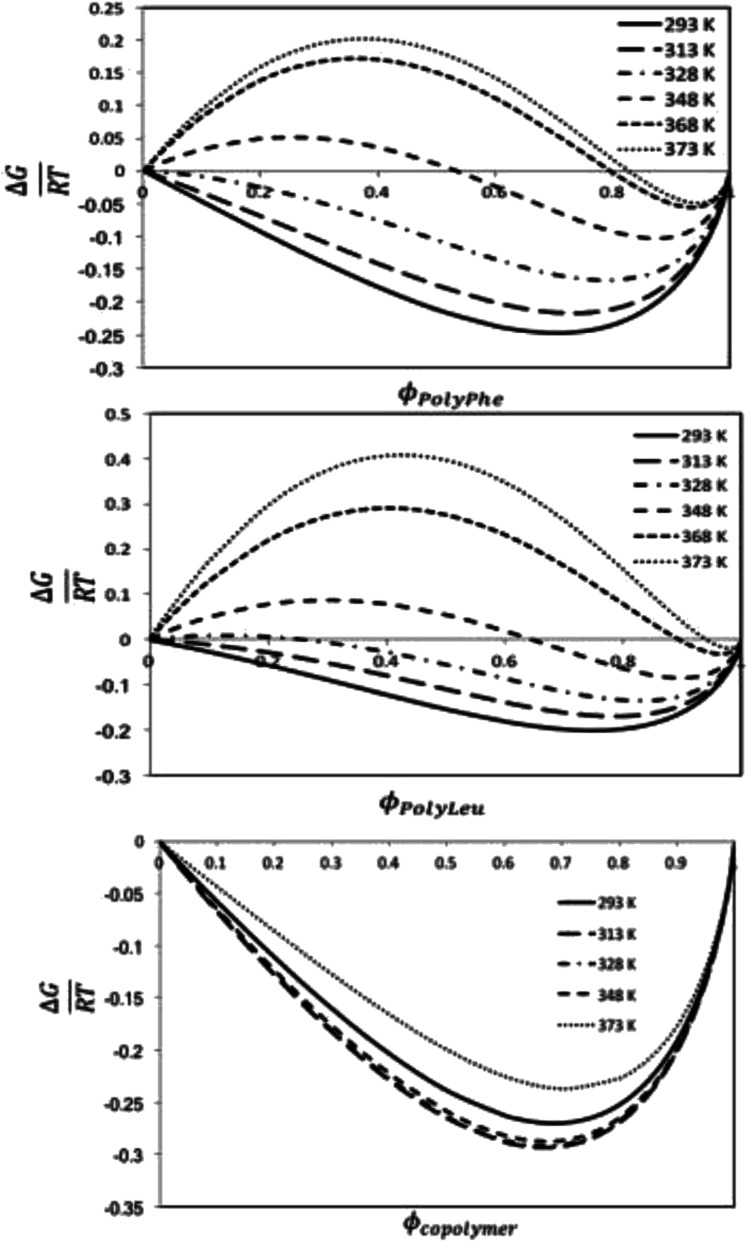
Calculated Δ*G* energy of mixing to polymer volume fraction at different temperatures relationships for the CF-poly(l-PheOEt) (top), CF-poly(l-LeuOEt) (center) and CF-poly(l-LeuOEt-*co*-l-PheOEt) (below) systems.

Our objective was to compute the Gibbs free energy mixtures of the mixtures composed of liquid CF-poly(l-PheOEt), CF-poly(l-LeuOEt) and CF-copolypeptide, relative to that of the two pure compounds by using the Flory–Huggins interaction parameter (*χ*_i–j_). This parameter relates to solubility parameters as, the solvent will be poorer for this polymer when *χ*_i–j_ increases, and on the contrary, a decrease in *χ*_i–j_ improves solubility of the polymer. Fig. 8 shows the calculated Δ*G* of mixing for the systems as a function of the volume fraction of the polypeptide. The calculations predict partial miscibility of this system and the appearance of phase separation at temperatures above 328 K and 313 K for CF-poly(l-PheOEt) and CF-poly(l-LeuOEt), respectively. This behavior can result from the decrease in the solubility parameter of the solvent at higher temperatures (Fig. 6). Nonetheless, at the operational temperature used in this study, the solutions are predicted to be thermodynamically miscible for all volume fractions of the polypeptides. Concerning the copolymer behavior, the calculated Gibbs energy of mixing is negative for all temperatures, which points out to spontaneous mixing concomitantly with the regular solution theory.

This behavior can arise from the lower polymerization degree, and consequently, the lower molar mass, obtained for the copolymer than for the two polymers. Additionally, density is lower for the copolymer than for polymers, indicating a higher free volume between the copolymer chains, allowing more sites available for interaction with the solvent. From solubility parameters, there is a more significant mismatch between solubility parameters values for this CF and the homopolypeptides than that observed for poly(l-LeuOEt-*co*-l-PheOEt), which also explains the better solubility of the copolymer in the solvent. Noteworthy, no experimental data on the phases behavior of these systems is available in the literature.

## Conclusions

Synthetic polypeptides using l-LeuOEt and l-PheOEt as model substrates has been successfully achieved using SC protease in the neat 1,1,1,2-tetrafluoroethane compressed to its liquid state. Comparative experiments with scCO_2_ solvent gave unsuccessful results owing to low polarity and the presence of carbamates. Product characterizations showed high molar mass polymers of up to 20 000 Da for poly-l-PheOEt. The enzyme showed significant affinity for the l-PheOEt monomer with up to a 1.8 : 1 (l-Phe : l-Leu) molar ratio of the copolypeptide. The computational studies based on molecular dynamics calculations assessed the solubility behavior of these systems by using the regular solution theory through the calculation of the Flory–Huggins solvent–polymer parameter. These calculations show that the copolymer has better miscibility with the organic CF than the polymers, which has been explained by the higher mismatch in the Hildebrand solubility parameters between the solvent and the polymers than between the solvent and the copolymer. The Gibbs energy of mixing calculations predict thermodynamically miscible liquid 1,1,1,2-tetrafluoroethane-copolypeptide mixtures for all the studied temperatures and volume fractions, whereas phase separation is expected to occur for this CF to homopolypeptide systems for some temperatures and polymer volume fractions.

## Experimental

### Materials

Sigma-Aldrich supplied l-leucine ethyl ester hydrochloride [l-LeuOEt·HCl] (≥99% purity), l-phenylalanine ethyl ester hydrochloride [l-PheOEt·HCl] (≥99% purity). Desalting proceeded by the preparation of 11 mL of potassium hydroxide (39 mmol) in solution, which was added to a 22 mL solution of the l-amino acid ethyl ester hydrochloride (26 mmol). After stirring for 1 h, the mixture was extracted four times with ethyl acetate (20 mL). The organic extracts were dried over calcium chloride and the solvent removed under reduced pressure to give a yellow liquid with a 60% yield. Sigma-Aldrich supplied SC (Subtilisin A serine S8 endoproteinase EC.3.4.21.62; 27 kDa) from *Bacillus licheniformis*. For enzyme activity, SC was incubated in liquid 1,1,1,2-tetrafluoroethane for 3, 6, 24 and 48 h, then dissolved (0.064 mg mL^−1^) in a solution (10 mM sodium acetate buffer with 5 mM calcium acetate, pH 7.5). The proteolytic assay proceeded as follows: one unit (U) of protease activity was equivalent to 1 μmol of l-tyrosine (JT Baker Mexico) liberated by the amount of enzyme per minute. Data is the result of three replicates and compared to a control without CF treatment. The U for the subtilisin Carlsberg used in this work was 8.39 ± 0.5 mmol min^−1^ mg^−1^. CEI de Mexico SA de CV (Mexico) supplied 1,1,1,2-tetrafluoroethane Dupont Suva-R134a 60 kg cylinder.

### Experimental assessment of the solubility of monomers in liquid 1,1,1,2-tetrafluoroethane and compressed carbon dioxide

l-LeuOEt and l-PheOEt substrates, individually (6 mmol) or as an equimolar mixture of both (3 mmol) were placed in a st-316 cylindrical view cell (40 mL) equipped with three sapphire windows, magnetic stirrer, and an external ceramic heating jacket. Experiments were conducted with and without enzyme. The CF was feed into the vessel by an ISCO 160XD Syringe pump under stirring at 25 bar and 313.15 K. The complete solubility of monomers was assessed visually throughout the sapphire windows. Identical experiments were carried out with scCO_2_ (313.15 K and 120 bar).

### Enzymatic polypeptide syntheses in liquid 1,1,1,2-tetrafluoroethane

In a typical experiment, SC (110 U), an amino acid ethyl ester (6 mmol) or the mixture (3/3 mmol) and a magnetic bar were placed inside a high-pressure-resistant st-316 cylindrical vessel (40 mL) equipped with two Swagelok (USA) high-pressure valves and an external ceramic heating jacket. Co-polymerization reactions were identically carried out by adding 6 mmol of a 1 : 1 equimolecular mixture of both amino acid ethyl esters. Then, the CF was feed into the vessel by the ISCO 160XD Syringe pump until the desired pressure (25 bar) was achieved at 313.15 K. The reaction mixture was magnetically stirred for 3, 6, 24 and 48 h. After each time, the vessel was cooled to room temperature and pressure relieved to atmospheric pressure and the contents were collected in deionized water (18.2 mΩ cm @ 313.15 K). Samples were centrifuged at 7000 × *g* for 20 min, and the supernatant was recovered and lyophilized to obtain the polypeptides as white powders. The supernatant aqueous extract of poly(l-PheOEt) were directly used in CD analyses. Product yields were calculated gravimetrically as a percentage from initial amino acid or the mixture of amino acids ethyl esters mass to product mass.

Poly(l-LeuOEt): ^1^H NMR (400 MHz, DMSO-d_6_/7.5% CF_3_COOH *δ* ppm): 0.89 (dd, *J* = 6.6, 3.4 Hz, 270H) (a), 1.22 (t, *J* = 7.1 Hz, 3H) (b), 1.54–1.68 (m, *J* = 7.0 Hz, 90H) (c), 1.76 (dp, *J* = 13.3, 6.7 Hz, 45H) (d), 3.83 (d, *J* = 7.5 Hz, 45H) (e), 4.20 (dd, *J* = 7.1, 2.8 Hz, 2H) (f), 8.33 (d, *J* = 16.4 Hz, 77H) (g). Poly(l-PheOEt): ^1^H NMR (400 MHz, DMSO-d_6_/7.5% CF_3_COOH *δ* ppm): 1.07 (t, *J* = 7.1 Hz, 3H) (a), 3.02–3.19 (m, 152H) (b), 4.10 (dd, *J* = 7.1, 2.0 Hz, 2H) (c), 4.11–4.22 (m, 76H) (d), 7.20–7.35 (m, 76H) (e), 8.26–8.43 (m, 97H) (f). Poly(l-LeuOEt-*co*-l-PheOEt): ^1^H NMR (400 MHz, DMSO-d_6_/7.5% CF_3_COOH *δ* ppm): 0.89 (dd, *J* = 6.5,3.7 Hz, 132H) (a), 1.08 (t, *J* = 7.1 Hz, 3H) (b), 1.53–1.70 (m, 44H) (c), 1.75 (dq, *J* = 13.3, 6.7 Hz, 22H) (d), 3.10 (d, *J* = 6.5 Hz, 58H) (e), 3.83 (d, *J* = 7.4 Hz, 22H) (f), 4.09 (dd, *J* = 7.1, 2.0 Hz, 2H) (g), 4.11–4.21 (m, 29H) (h), 7.16–7.37 (m, 145H) (i), 8.18–8.76 (m, 576H) (j).

Control reactions for both operating conditions were carried out during 24 h without the biocatalyst. Identical experiments were conducted with supercritical CO_2_ (313.15 K, 120 bar).

### Characterization of polypeptides

Nuclear Magnetic Resonance (NMR) ^1^H NMR, ^13^C NMR, and 2D NMR spectra were recorded on a Varian VNMR and MR (400 MHz) spectrometer at room temperature controlled with VNMRS. NMR experiments were performed in deuterated dimethyl sulfoxide (DMSO-d_6_) containing 7.5% deuterated trifluoroacetic acid (TFA-d) at 60 mg mL^−1^. Methyl end group was selected to determine molar mass compared to assigned signals of the repeat units in ^1^H NMR spectra. l-PheOEt to l-LeuOEt ratios in the co-polypeptides were calculated according to the integration of the characteristic signals of each repeat unit on the ^1^H NMR spectra. ATR-FTIR spectra were recorded in a Perkin-Elmer Spectrum 400 spectrometer from 400 to 4000 cm^−1^. Powder X-ray diffraction (PXRD) spectra were acquired in the 3° ≤ 2*θ* ≤ 60° range with a Bruker D8 Advance diffractometer using Cu Kα radiation (*λ* = 1.5406 Å) and goniometer speed of 0.5°(2*θ*) min^−1^. Crystallinity percentages were obtained by integration of the corresponding crystalline and amorphous areas on the spectra. Circular dichroism (CD) spectrum was recorded on a JASCO J-715 Spectropolarimeter (Jasco Inc., USA) for an aqueous extract of poly(l-PheOEt) at room temperature using a 1 mm path length cell. Spectra were acquired from 190 to 260 nm, and ellipticity is reported as mdeg. UV-Vis spectra for poly(l-PheOEt) and l-PheOEt were recorded in a Lambda 2S UV-Vis spectrophotometer (Perkin Elmer Inc., USA) at room temperature in a 1 cm path length cell, from 500 to 200 nm.

### Statistical analysis

Statistical analysis was performed by ANOVA test to compare the means of all times of reaction, and Tukey's test was applied to determine a statistically significant difference between groups. The test confidence level was set at 95% (*p* < 0.05) (OriginPro software, Microcal Corp., USA).

### Computational modeling of solvent–product interactions

MD calculated density, solubility parameters and Cohesive Density Energy (CED) for the compressed 1,1,1,2-tetrafluoroethane, poly(l-PheOEt), poly(l-LeuOEt) and the copolypeptides at different temperatures and pressures. For these calculations, a box containing liquid 1,1,1,2-tetrafluoroethane and the polymers were simulated at a fixed temperature and pressure in the NPT ensemble. The degree of polymerization (DP) during molecular modeling was set to the experimental value for 24 h of reaction. Geometries for all molecules under study were initially optimized at the B3LYP/DNP level of theory. The hybrid density functional (B3LYP) and the double numerical with dynamic nuclear polarization (DNP) basis set within the DFT was carried out by the software package DMol3 in the Materials Studio suite from Biovia. Given the size of the polymers treated, the approximate atomic point charges were determined by the Gasteiger method. Simulated boxes for MD calculations were set with periodic boundaries in all directions containing a total of 500 molecules for the CF, 10 molecules in the case of poly(l-PheOEt) and poly(l-LeuOEt) and 8 molecules for poly(l-LeuOEt-*co*-l-PheOEt). The ensemble used was the isobaric-isothermal ensemble (NPT). A typical simulation box for both compounds is shown in the ESI File 1.† MD simulations were performed as follows, NPT equilibration and density production runs of 1 200 000 steps were performed by using the Forcite module from the Materials Studio suite from Biovia. All runs were performed with a time step of 1 fs. L-J and the coulombic cut-off was set to 15 Å. Calculations were performed in the Occigen super-calculator (BullX SCS6) of the CINES (Centre Informatique National de l’Enseignement Superior) in Montpellier, France. Density of liquid 1,1,1,2-tetrafluoroethane (313.15 K, 25 bar) and polypeptides were calculated by MD simulations using the Forcite module with the COMPASSII (Condensed-phase Optimized Molecular Potentials for Atomistic Simulation Studies) force field,^36^ which is a force field optimized for condensed-phase systems where the parameters are derived from quantum mechanics data and calculations as well as from fittings of the experimental condensed phase properties. During polymers and copolymer construction, polymerization degree was fixed to 134 in the case of poly(l-PheOEt) and 108 in the case of poly(l-LeuOEt). The Leu/Phe ratio for the copolymer was 1.05, and the polymerization degrees were fixed to 29 for poly(l-LeuOEt) and 31 for poly(l-PheOEt). The molar mass of the copolypeptide was fixed to 8044 Da while for poly(l-LeuOEt) and those for poly(l-PheOEt) were 12 366 Da and 19 923 Da, respectively. Next, the cohesive energy density (CED, eqn (1)) was determined by sampling the system and collecting data from the last 400 ps from the production runs.1
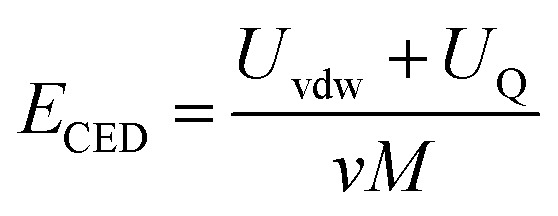
where *U*_vdW_ and *U*_Q_ are the van der Waals and electrostatic energy, respectively. The solubility parameter of each component (*δ*) can be expressed by eqn (2)2
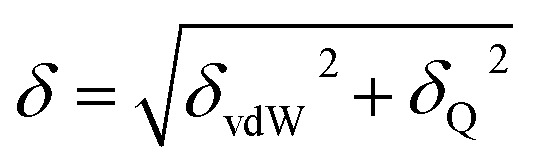
where *δ*_vdW_ and *δ*_Q_ represent the contributions from van der Waals forces and electrostatic interactions, respectively, and calculated from eqn (3) and (4)3
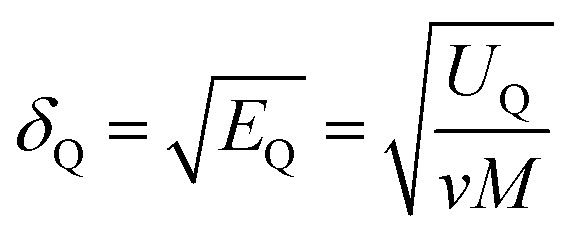
4
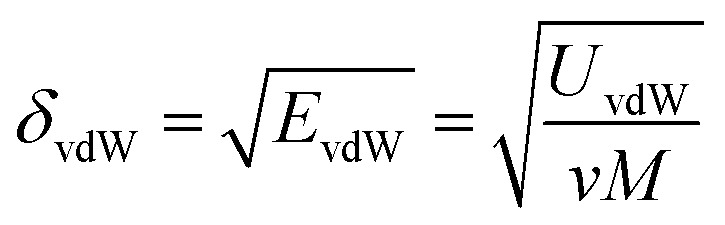
where *E*_Q_ is the electrostatic energy density and *E*_vdW_ is the van der Waals energy density; the solubility parameter has dispersion and electrostatic components, which together with hydrogen bonding are frequently used to study and explain solvency phenomena.

The solubility parameter of each component (*δ*_i_) was readily calculated from CED by equation (5)5
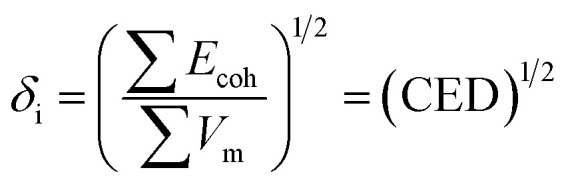


#### Flory–Huggins theory and energy of mixing

The Flory–Huggins interaction parameters for liquid 1,1,1,2-tetrafluoroethane and the enzymatically synthesized polypeptides at different *T* and *P* were obtained using eqn (6) (ref. 37)6
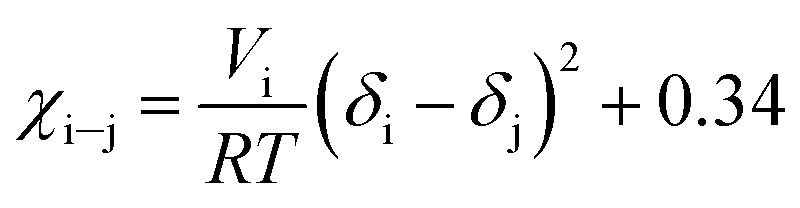
where *χ*_i–j_ is the solvent–polymer interaction parameter; *V*_i_ is the molar volume of the solvent; *R* is the gas constant; *T* is the absolute temperature and *δ*_i_ and *δ*_j_ are the solubility parameters for the solvent and the polymer, respectively.

The Gibbs energies of mixing for the CF-poly(l-PheOEt), CF-poly(l-LeuOEt) and CF-copolypeptide systems were calculated as a function of the volume fraction of the polymer at different temperatures by eqn (7)7
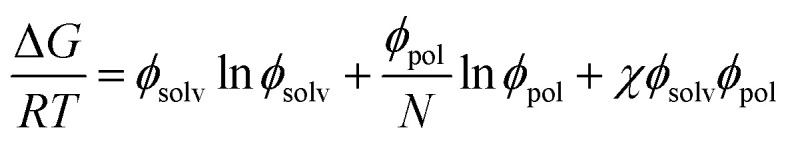
where *ϕ* represents the volume fraction of the solvent or the polymer and *N* the polymerization degree and *χ* the Flory–Huggins interaction parameter. For the calculation concerning the polymer, *N* was fixed to 30, as experimentally polymerizations degrees obtained were 29 for poly(l-LeuOEt) and to 31 for poly(l-PheOEt).

## Conflicts of interest

There are no conflicts to declare.

## Supplementary Material

RA-008-C8RA06657D-s001
