# Supplementary material for: Chemoenzymatic synthesis of polypeptides in neat 1,1,1,2-tetrafluoroethane solvent
Source: RSC Adv. 2018 Oct 22;8(63):35936–45. doi: 10.1039/c8ra06657d (PMC9088702; doi:10.1039/c8ra06657d)
Supplement: RA-008-C8RA06657D-s001 [file RA-008-C8RA06657D-s001.pdf]

Electronic Supplementary Material (ESI) for RSC Advances.
This journal is © The Royal Society of Chemistry 2018

Supplemental information 1: Molecular Modelling Typical box for a) liquid 1,1,1,2-tetrafluoroethane and b) poly(L-PheOEt).

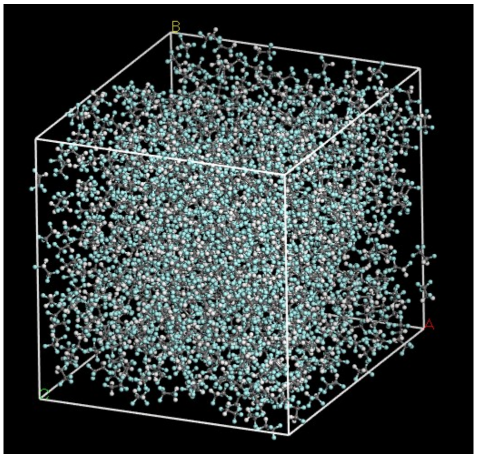

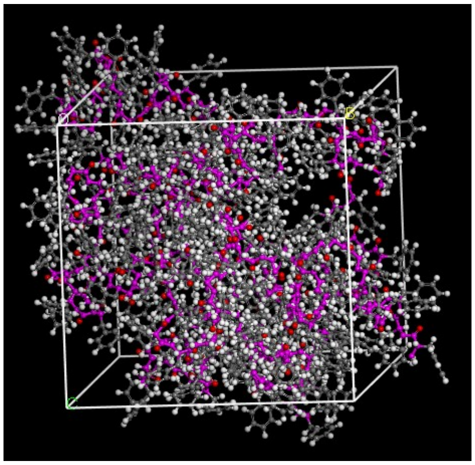

Supplemental information 2: representative 1H NMR spectrum of the carbamate product formed when attempted polymerization reaction in $scCO_2$

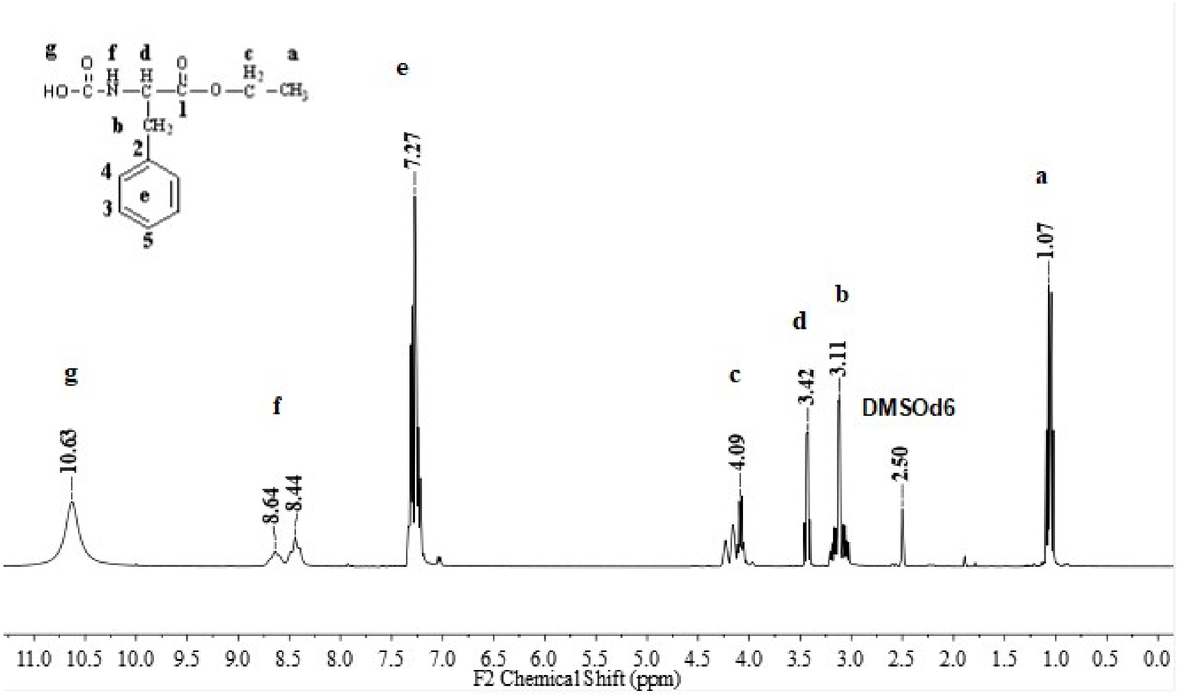

Supplemental information 3: NMR analyses of the products

Representative 1H NMR (A) and 13C NMR (B) spectra protease Subtilisin Carlsberg-mediated poly(L-LeuOEt) in liquid 1,1,1,2-tetrafluoroethane (40 °C, 25 bar)

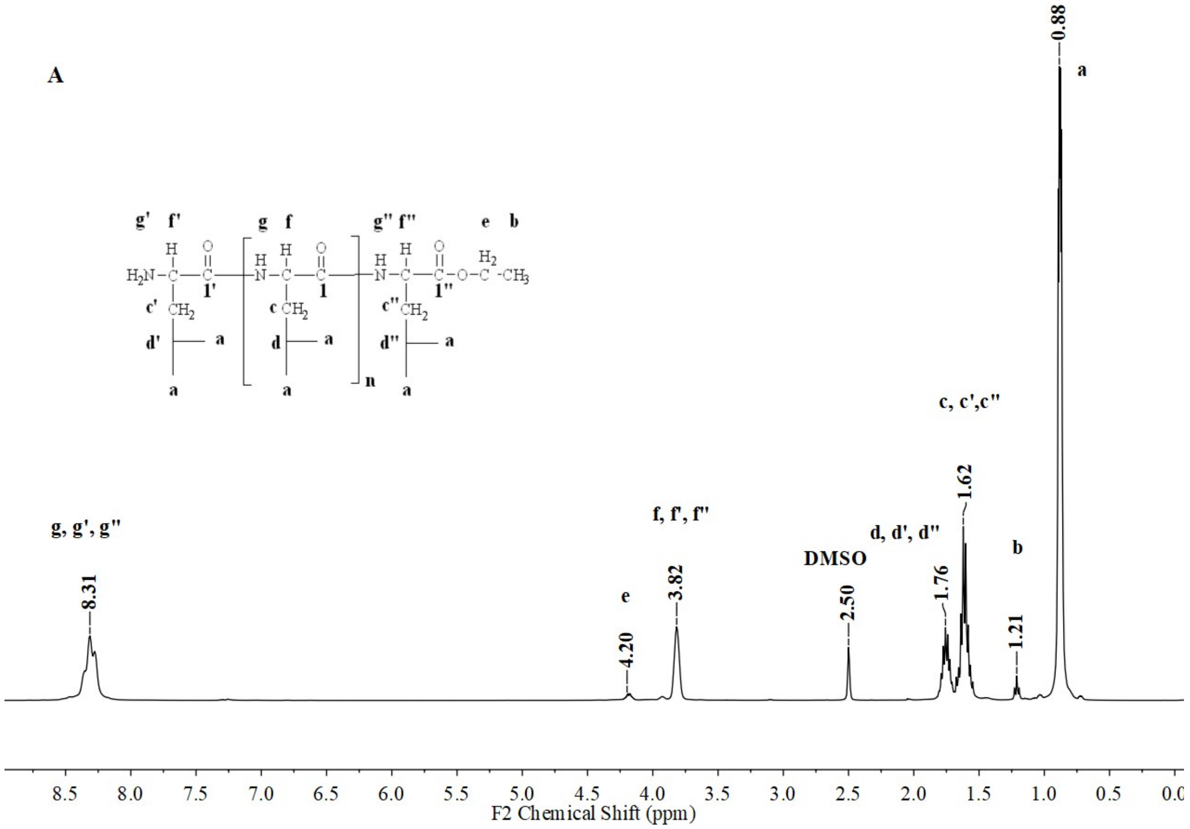

B

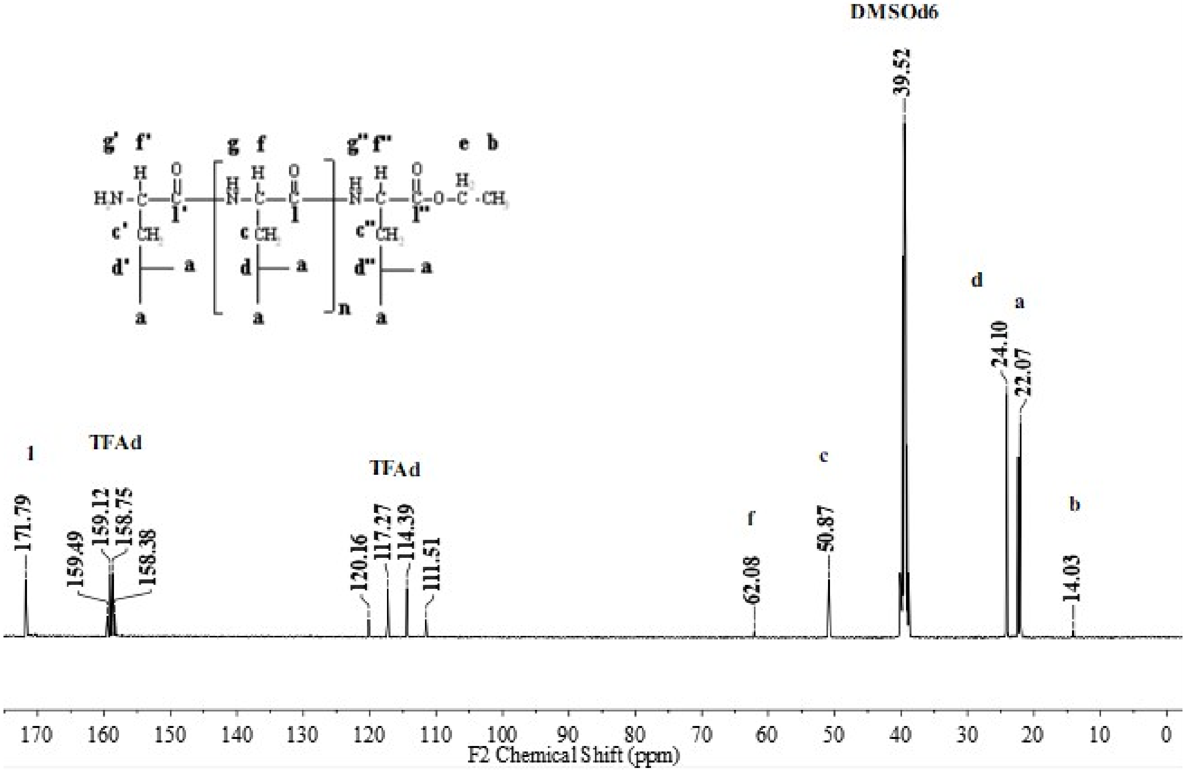

DMSOd6
39.52
d
a
24.10
22.07
1
TFAd
TFAd
c
b
f
171.79
159.49
159.12
158.75
158.38
120.16
117.27
114.39
111.51
62.08
50.87
14.03
170 160 150 140 130 120 110 100 90 80 70 60 50 40 30 20 10 0
F2 Chemical Shift (ppm)

Representative HMBCAD (A) and COSY (B) spectra for protease Subtilisin Carlsberg-mediated Poly(L-LeuOEt) in liquid 1,1,1,2-tetrafluoroethane (40 °C, 25 bar)

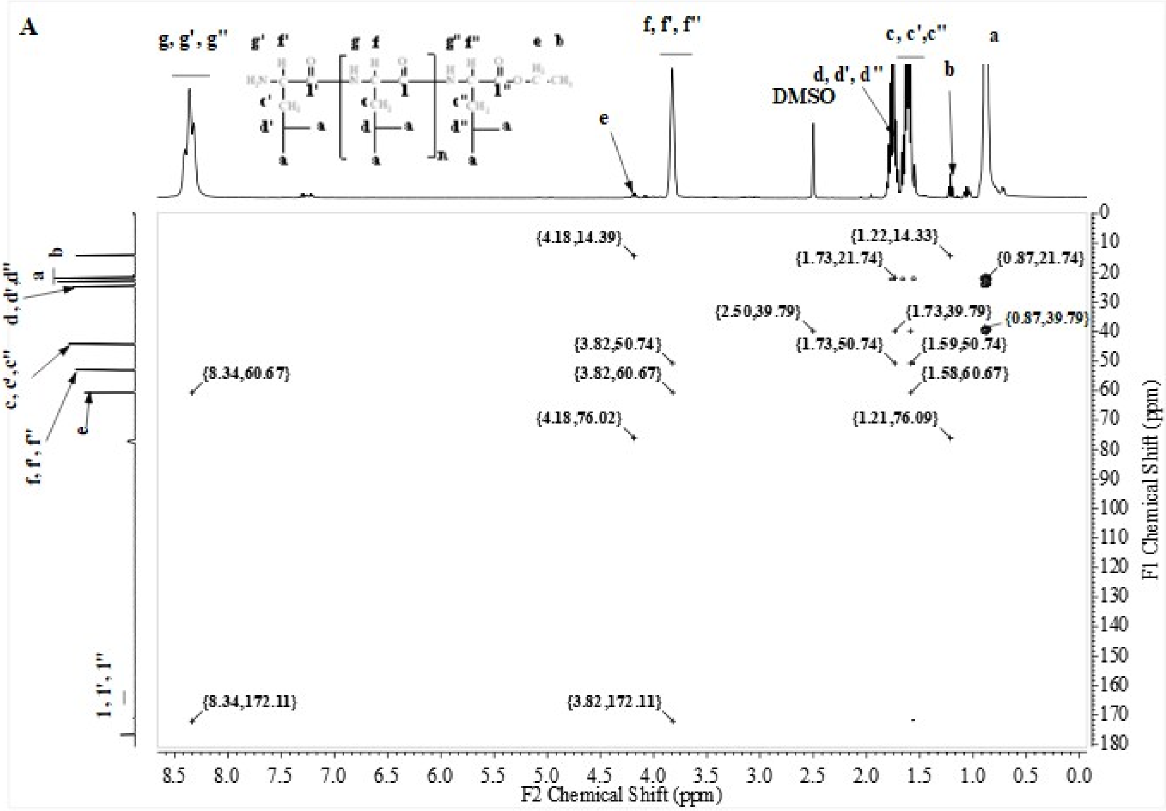

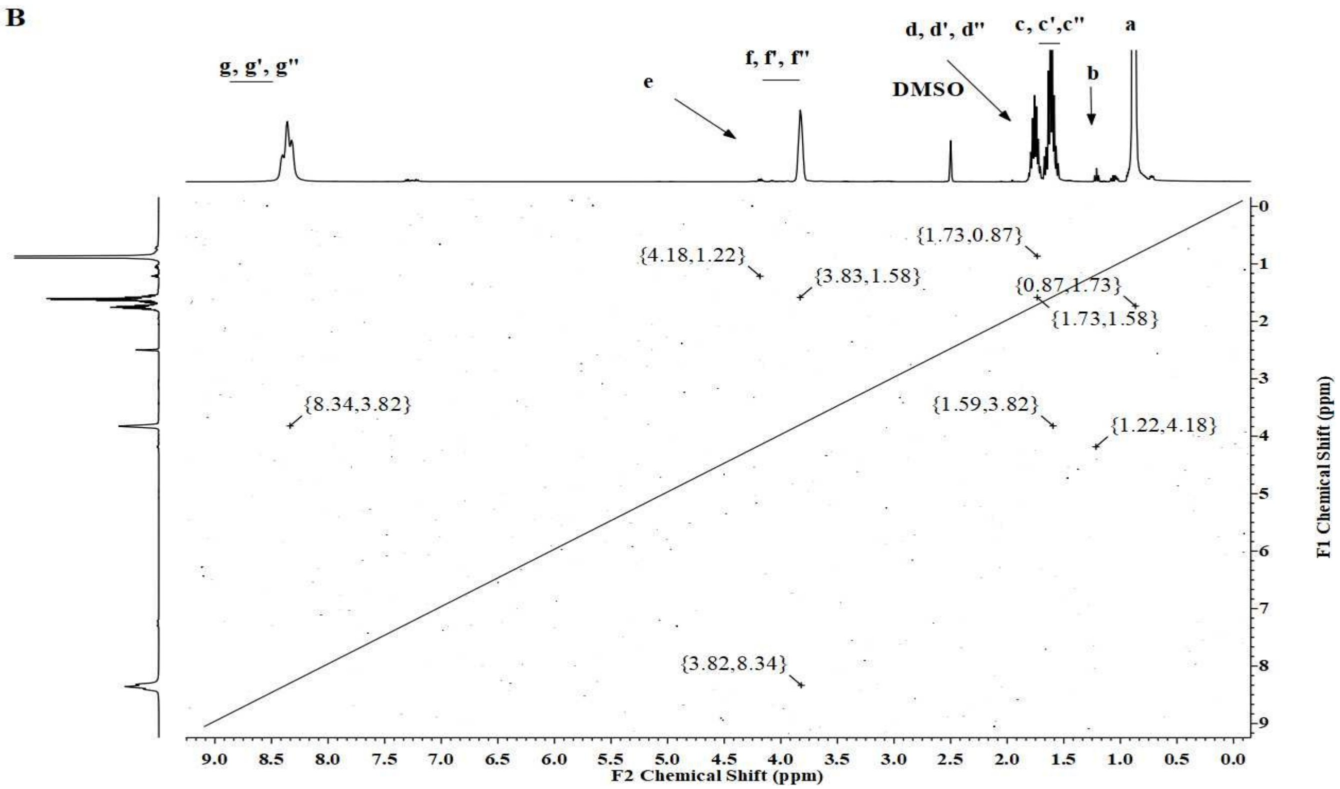
B
g, g', g''
e
f, f', f''
DMSO
d, d', d''
c, c',c''
b
a
{1.73,0.87}
{4.18,1.22}
{3.83,1.58}
{0.87,1.73}
{1.73,1.58}
{8.34,3.82}
{1.59,3.82}
{1.22,4.18}
{3.82,8.34}
F2 Chemical Shift (ppm)
F1 Chemical Shift (ppm)

Representative 1H NMR and 13C NMR spectra for protease Subtilisin Carlsberg-mediated Poly(L-PheOEt) in liquid 1,1,1,2-tetrafluoroethane (40 °C, 25 bar)

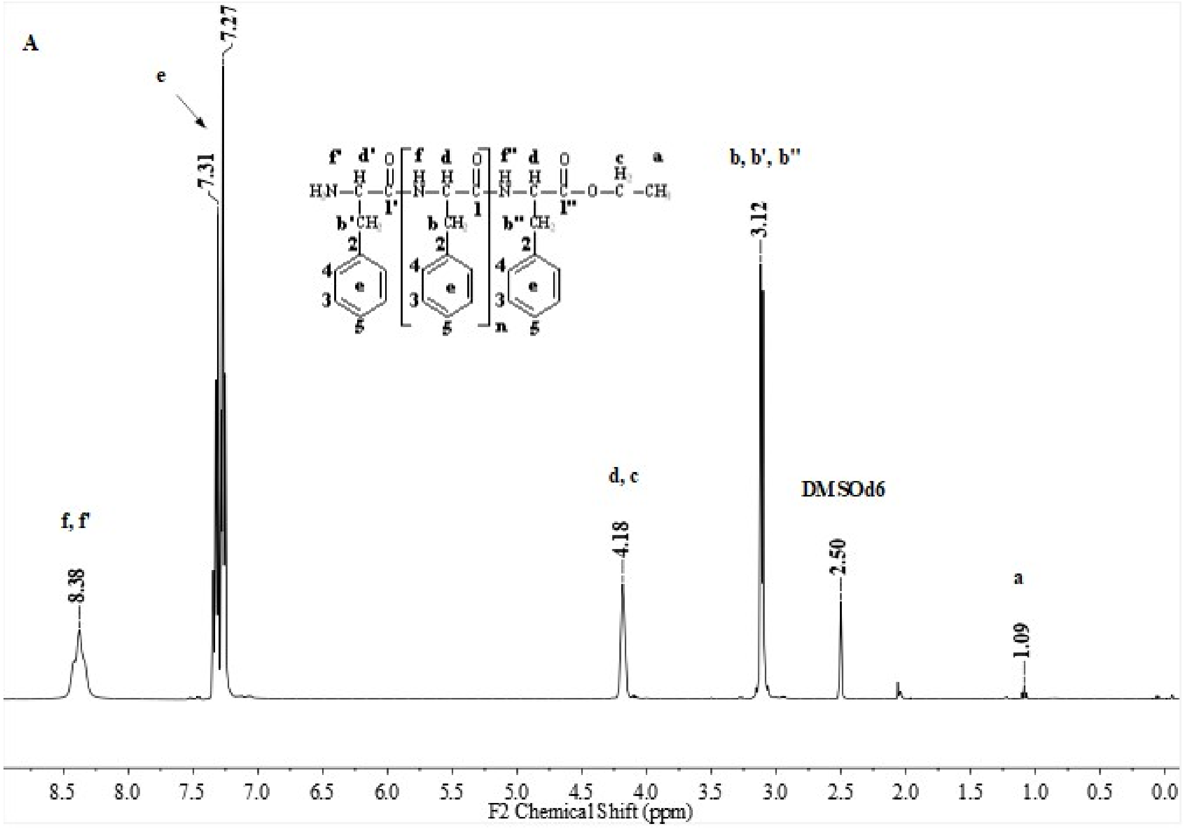

B

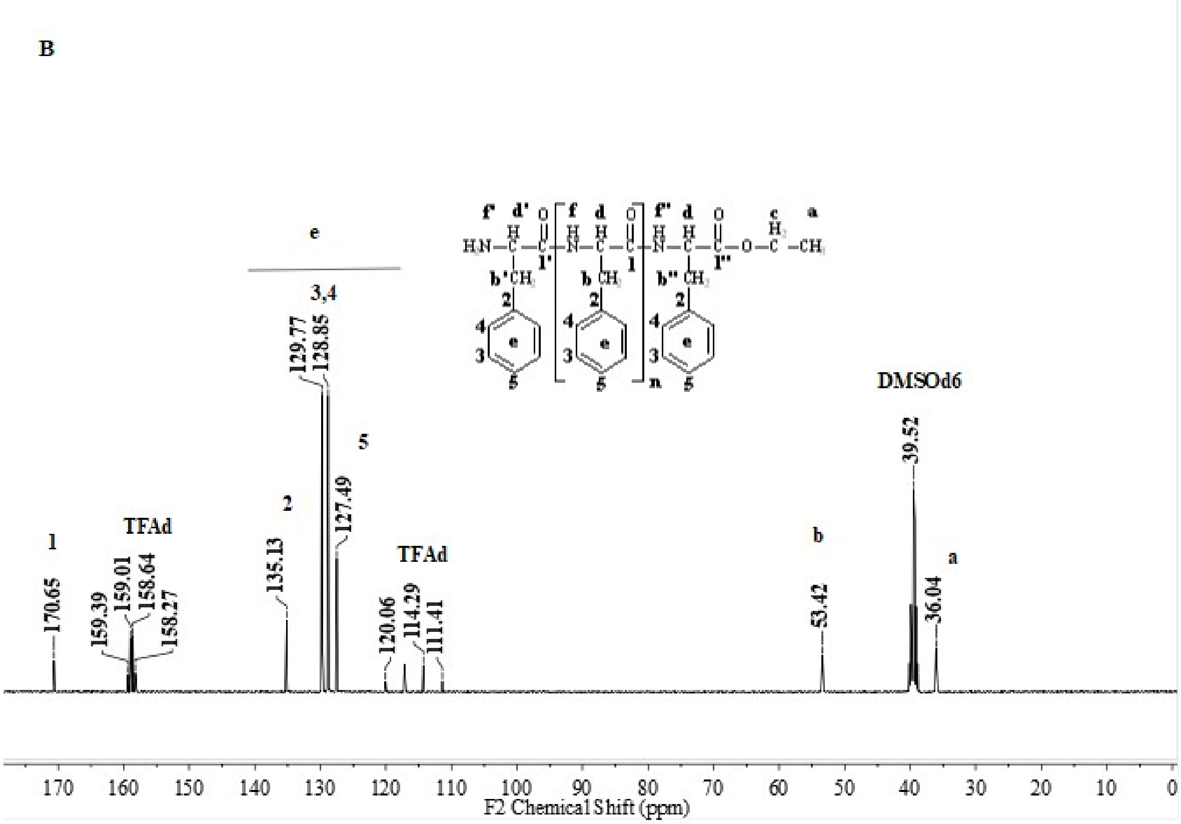

e
3,4
129.77
128.85
5
127.49
2
135.13
1
170.65
TFAd
159.39
159.01
158.64
158.27
TFAd
120.06
114.29
111.41
DMSOd6
39.52
b
53.42
a
36.04
170 160 150 140 130 120 110 100 90 80 70 60 50 40 30 20 10 0
F2 Chemical Shift (ppm)

Representative HMBCAD (A) and COSY (B) spectra for protease Subtilisin Carlsberg-mediated Poly(L-PheOEt) in liquid 1,1,1,2-tetrafluoroethane (40 °C, 25 bar)

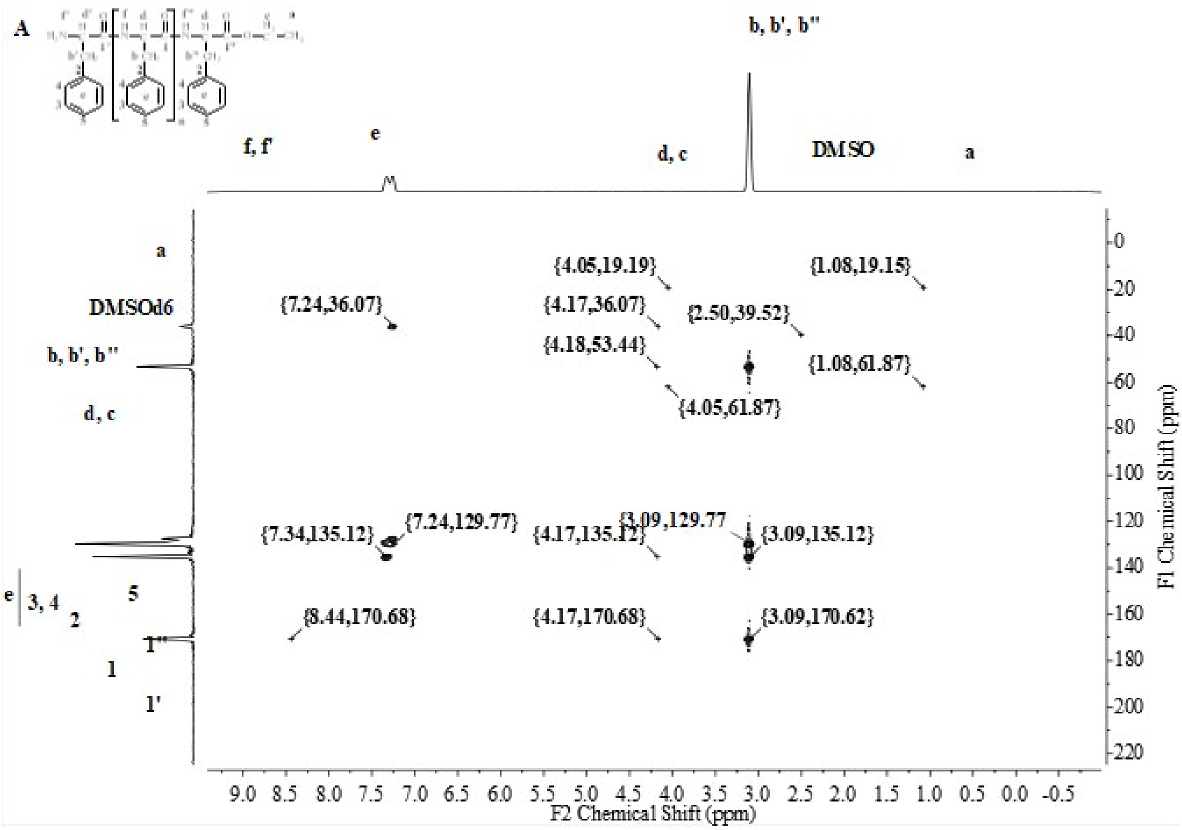

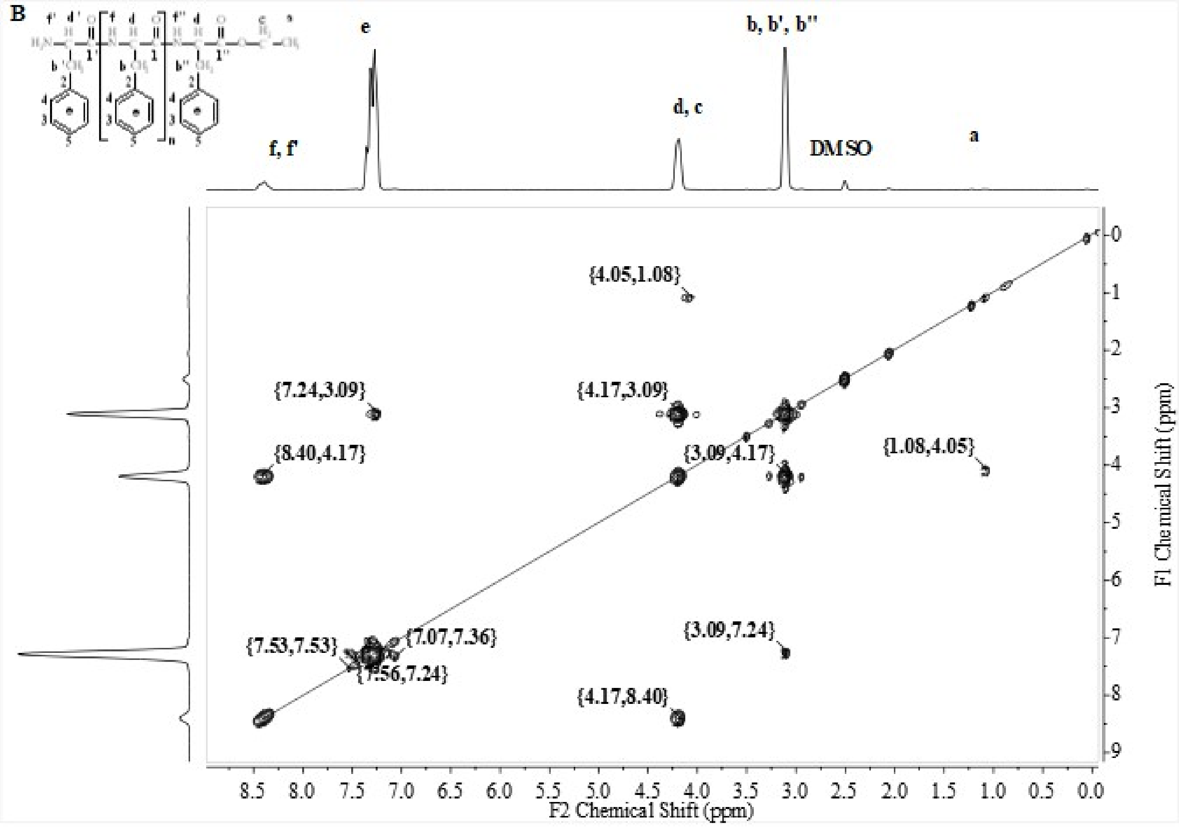

B
e
b, b', b''
d, c
f, f'
DMSO
a
{4.05,1.08}
{7.24,3.09}
{4.17,3.09}
{8.40,4.17}
{3.09,4.17}
{1.08,4.05}
{7.53,7.53}
{7.07,7.36}
{7.56,7.24}
{3.09,7.24}
{4.17,8.40}
F1 Chemical Shift (ppm)
F2 Chemical Shift (ppm)

Representative NMR spectra for protease of Subtilisin Carlsberg-mediated Poly(L-LeuOEt-co-L-PheOEt) in liquid 1,1,1,2-tetrafluoroethane (40 °C, 25 bar)

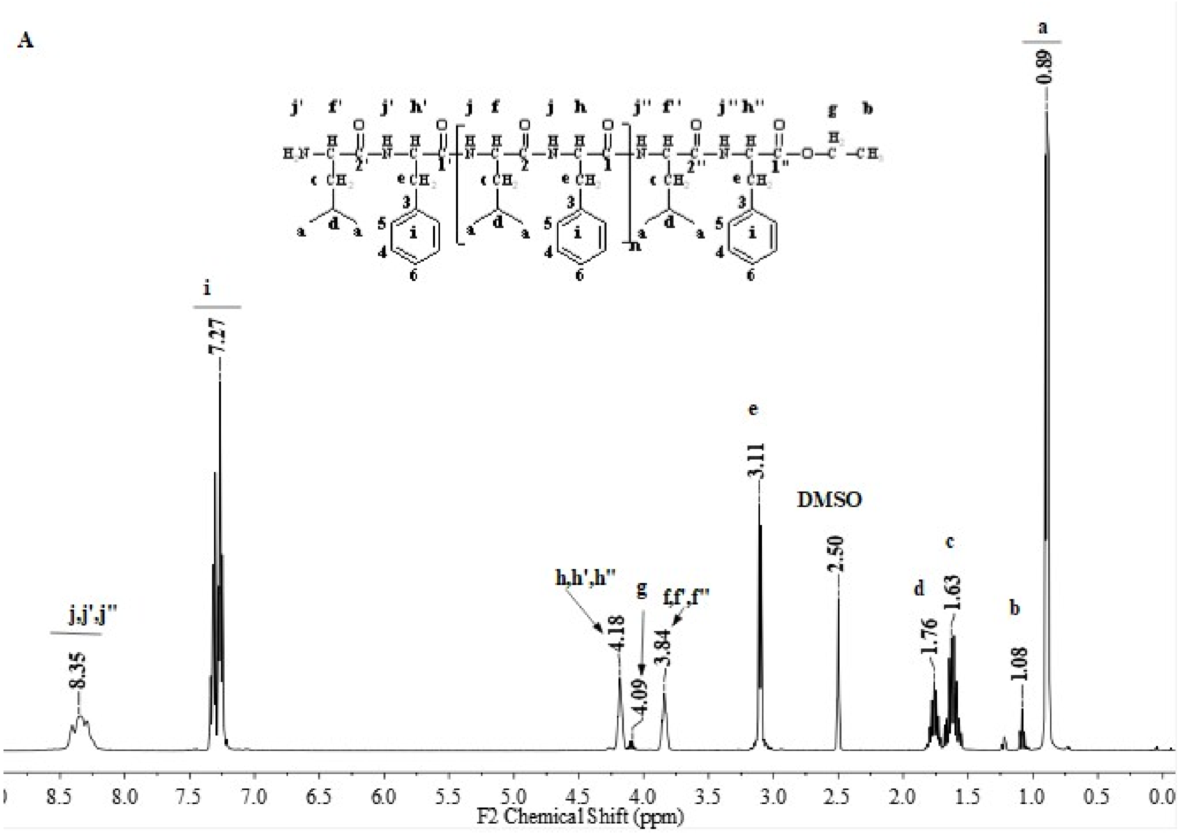

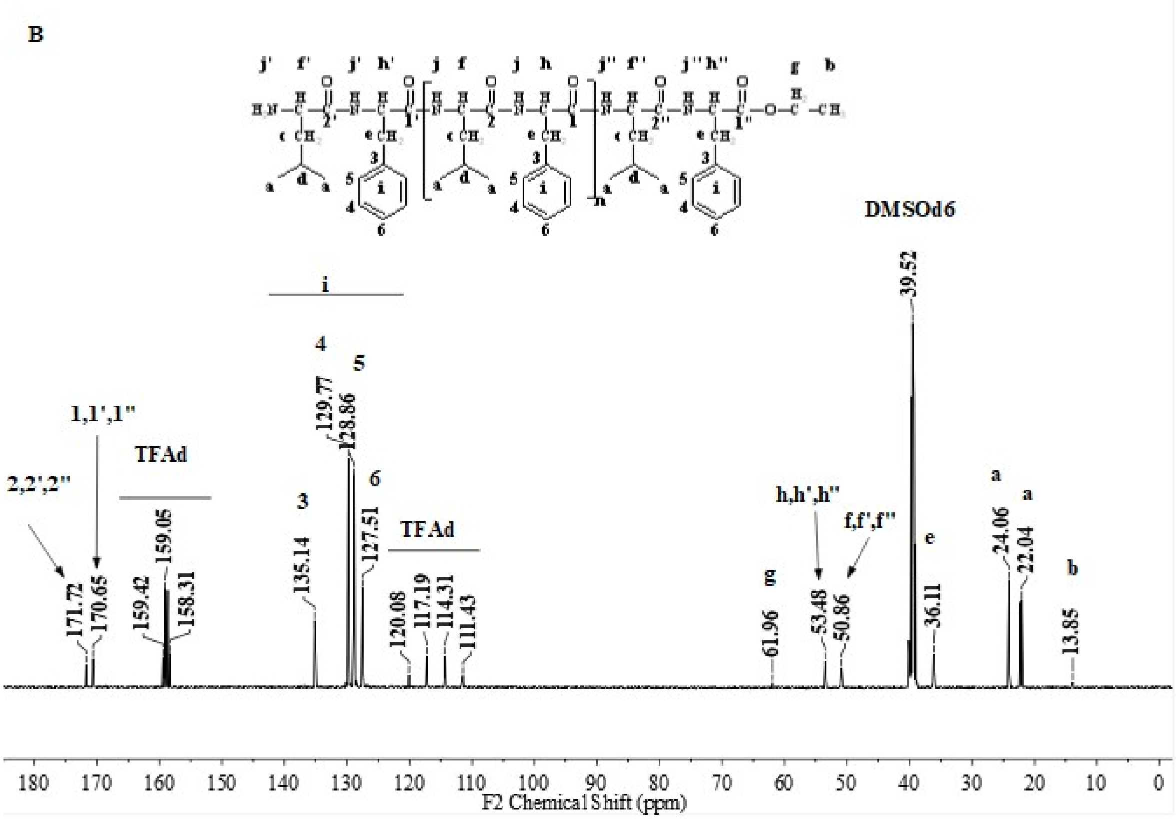

B
DMSOd6
39.52
i
4
129.77
128.86
5
6
127.51
3
135.14
1,1',1"
2,2',2"
171.72
170.65
TFAd
159.42
159.05
158.31
TFAd
120.08
117.19
114.31
111.43
g
61.96
h,h',h"
53.48
f,f',f"
50.86
e
36.11
a
24.06
a
22.04
b
13.85
180 170 160 150 140 130 120 110 100 90 80 70 60 50 40 30 20 10 0
F2 Chemical Shift (ppm)

Representative HMBCAD (A) and COSY (B) spectra for protease Subtilisin Carlsberg-mediated Poly(L-PheOEt-co-L-LeuOEt) in liquid 1,1,1,2-tetrafluoroethane (40 °C, 25 bar)

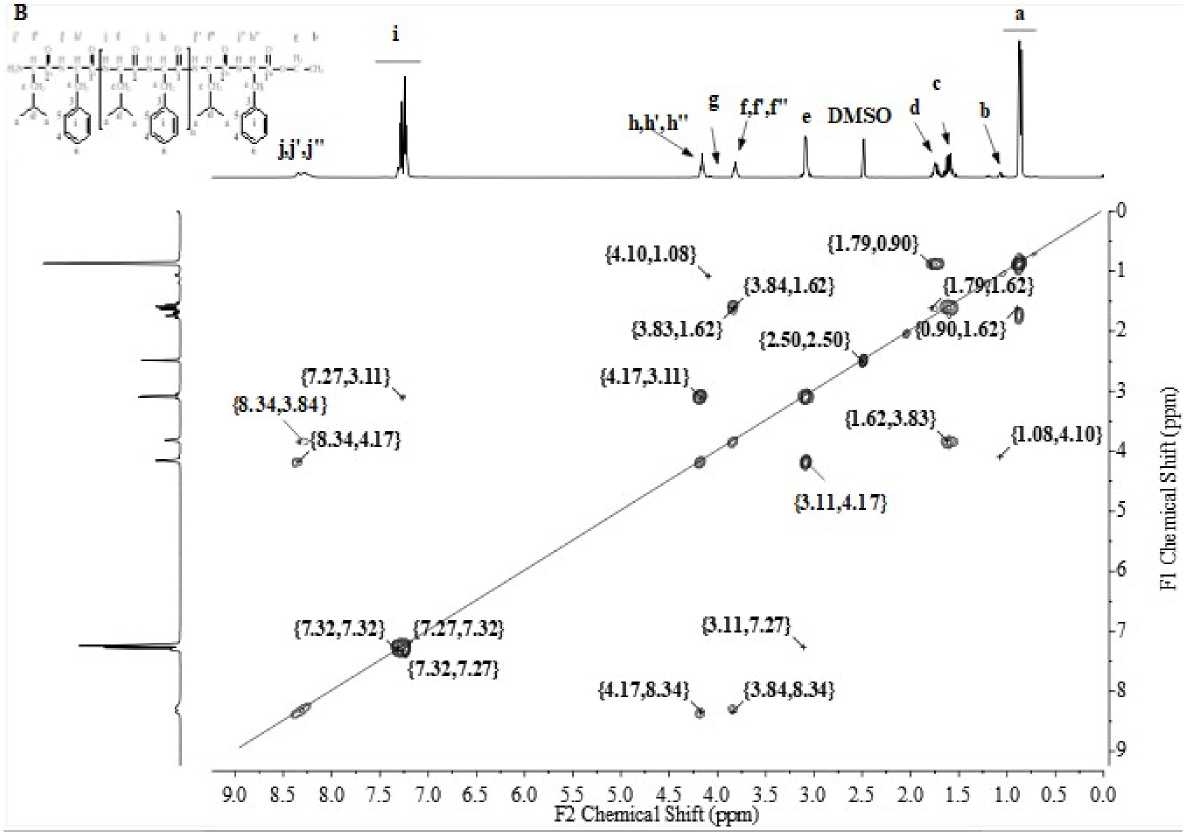

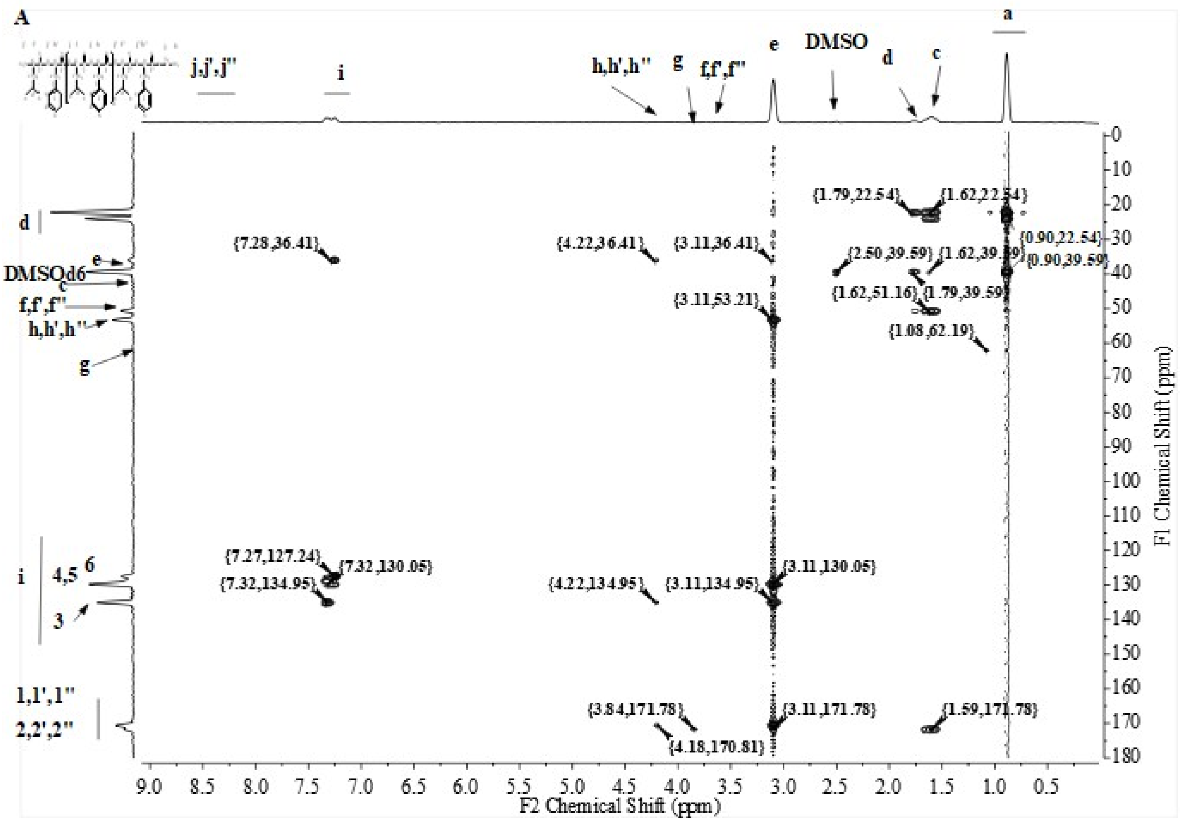

A
j,j',j''
i
h,h',h''
g
f,f',f''
e
DMSO
d
c
a
DMSOd6
4,5
6
3
1,1',1''
2,2',2''
{7.28,36.41}
{4.22,36.41}
{3.11,36.41}
{1.79,22.54}
{1.62,22.54}
{0.90,22.54}
{2.50,39.59}
{1.62,39.59}
{0.90,39.59}
{1.62,51.16}
{1.79,39.59}
{3.11,53.21}
{1.08,62.19}
{7.27,127.24}
{7.32,130.05}
{7.32,134.95}
{4.22,134.95}
{3.11,134.95}
{3.11,130.05}
{3.84,171.78}
{4.18,170.81}
{3.11,171.78}
{1.59,171.78}
F2 Chemical Shift (ppm)
F1 Chemical Shift (ppm)

Supplemental information 4. Representative ATR-FTIR spectra of each synthesized compounds. Poly(L-LueOEt) (A); Poly(L-PheOEt) (B) and Poly(L-LeuOEt-co-PheOEt) (C)

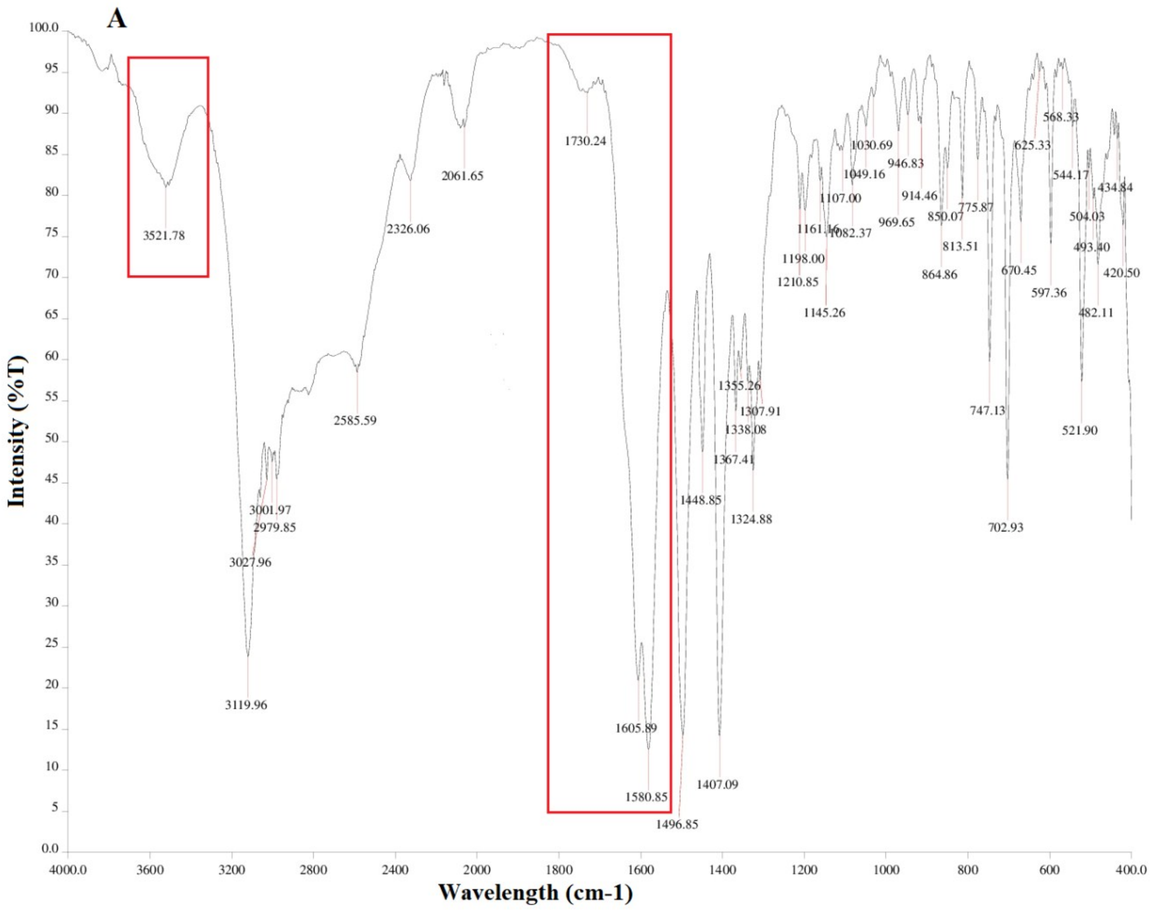

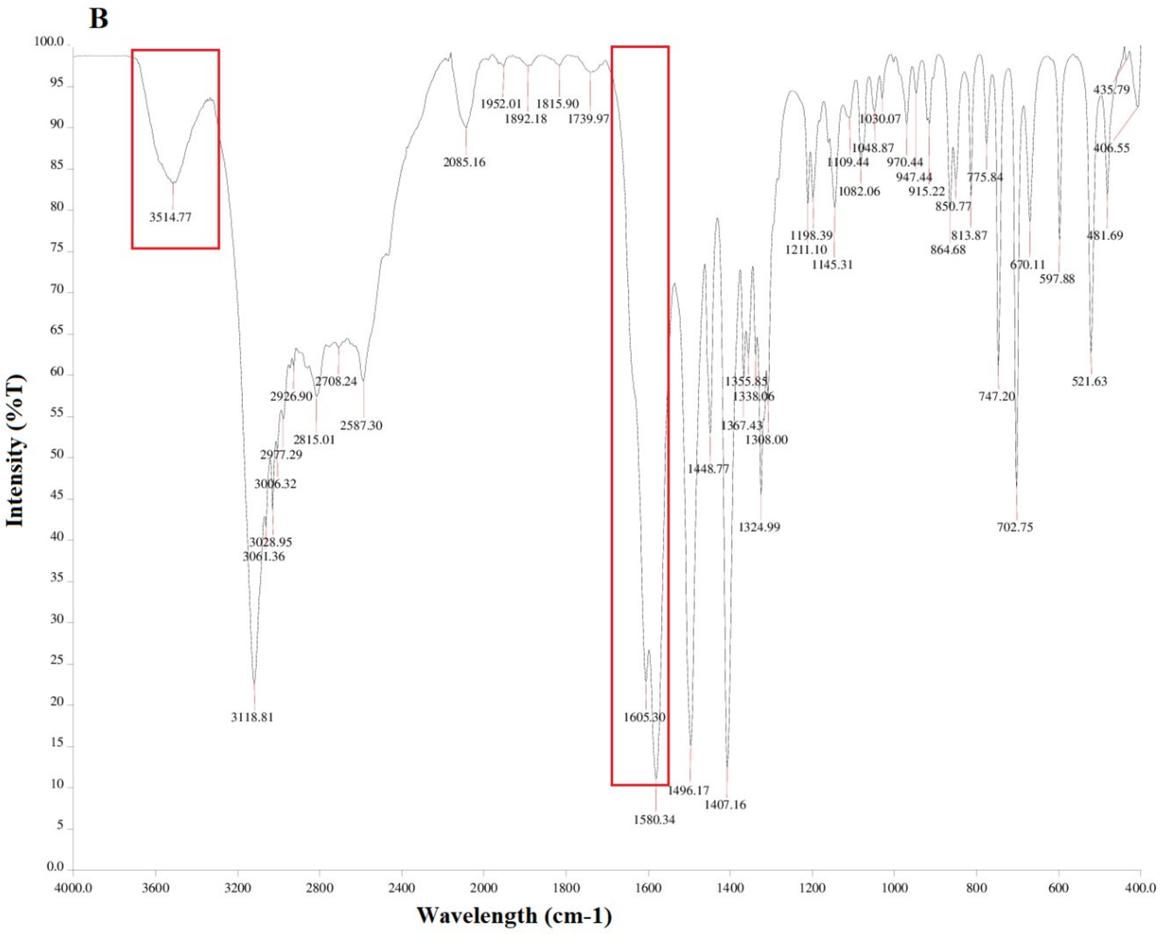

B
Intensity (%T)
Wavelength (cm-1)
3514.77
3118.81
3061.36
3028.95
3006.32
2977.29
2926.90
2815.01
2708.24
2587.30
2085.16
1952.01
1892.18
1815.90
1739.97
1605.30
1580.34
1496.17
1448.77
1407.16
1367.43
1355.85
1338.06
1324.99
1308.00
1211.10
1198.39
1145.31
1109.44
1082.06
1048.87
1030.07
970.44
947.44
915.22
864.68
850.77
813.87
775.84
747.20
702.75
670.11
597.88
521.63
481.69
435.79
406.55

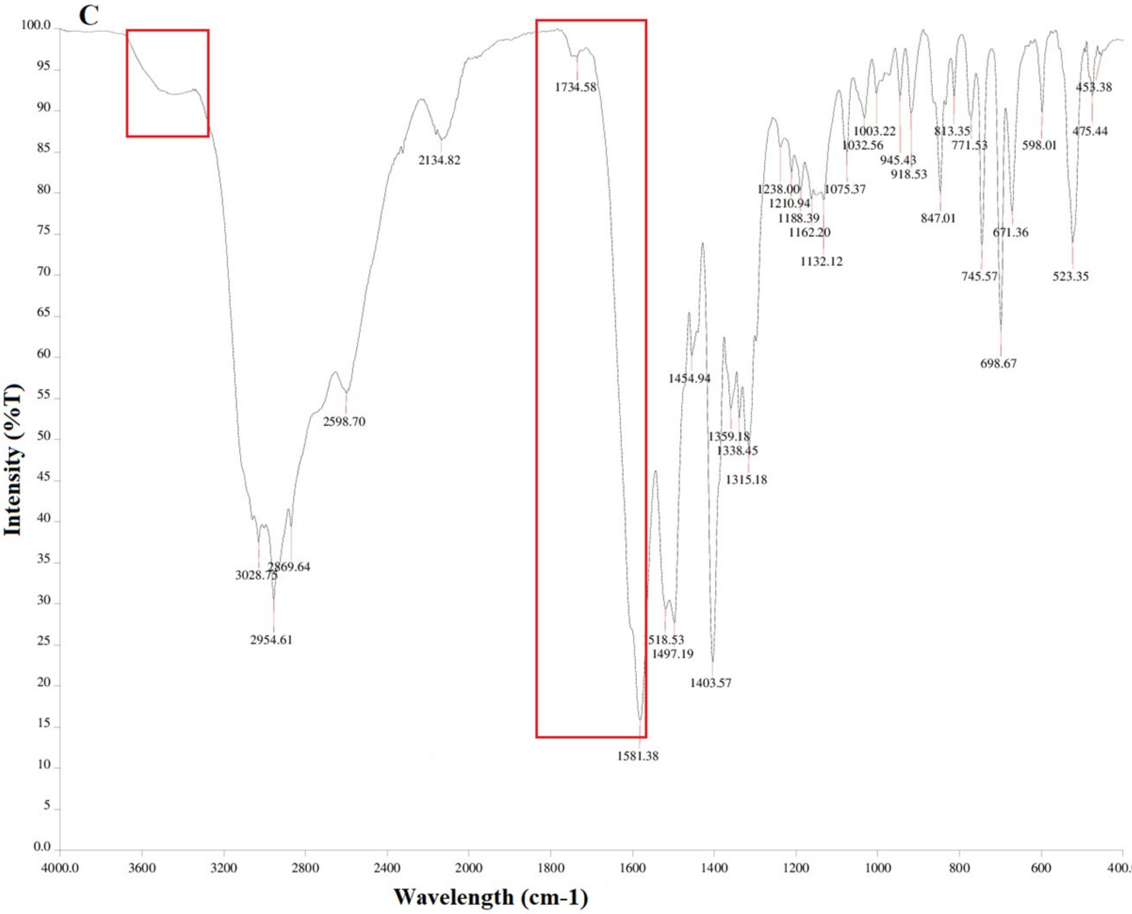
C
Intensity (%T)
Wavelength (cm-1)
3028.75
2954.61
2869.64
2598.70
2134.82
1734.58
1581.38
518.53
1497.19
1454.94
1403.57
1359.18
1338.45
1315.18
1238.00
1210.94
1188.39
1162.20
1132.12
1075.37
1032.56
1003.22
945.43
918.53
847.01
813.35
771.53
745.57
698.67
671.36
598.01
523.35
475.44
453.38

Supplemental information 5. UV spectra for poly(L-PheOEt) in solid line and L-PheOEt in dash line.

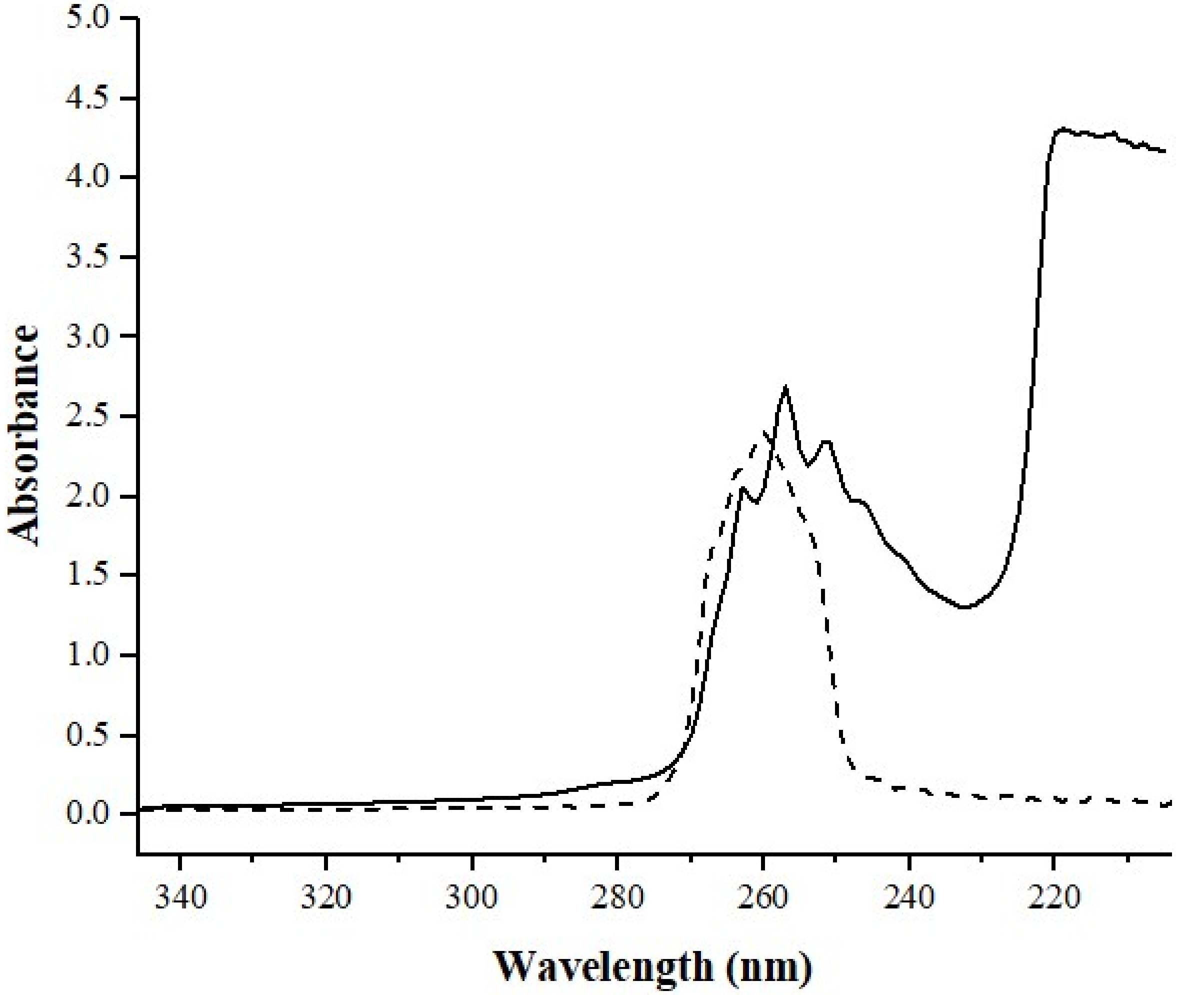

Supplemental information 6. Graphical representation of the results of control polymerization reactions without enzyme

Bars: average molar mass by 1H NMR (Da) of poly(L-LeuOEt) (A); poly(L-PheOEt) (B), poly(L-LeuOEt-co-L-PheOEt) (C) products in reactions carried out in liquid 1,1,1,2-tetrafluoroethane (40 °C and 25 bar) Line: average yield of weight of the polymers (%). Error bars represents the standard deviation of 3 replicates.

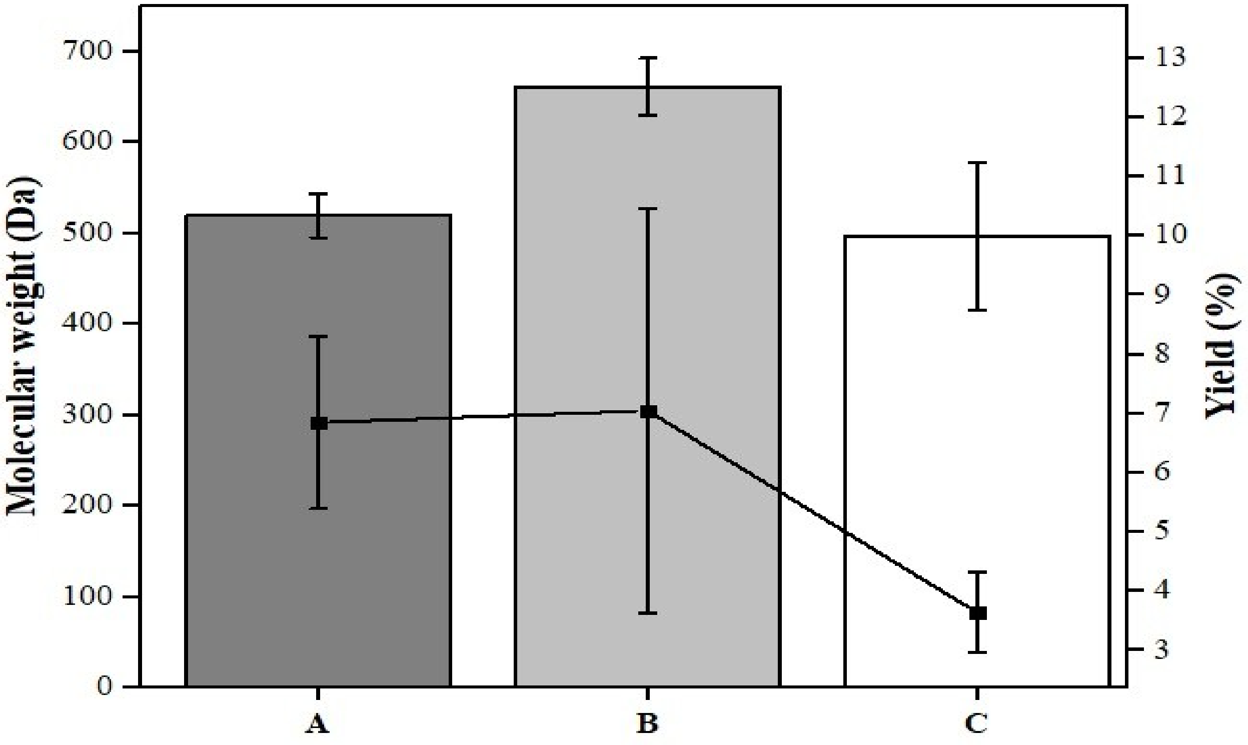

Supplemental information 7. PXRD diffractograms for the poly(L-PheOEt) (A), poly(L-LeuOEt) (B), and poly(L-LeuOEt-co-L-PheOEt) (C) at different reaction times (h).

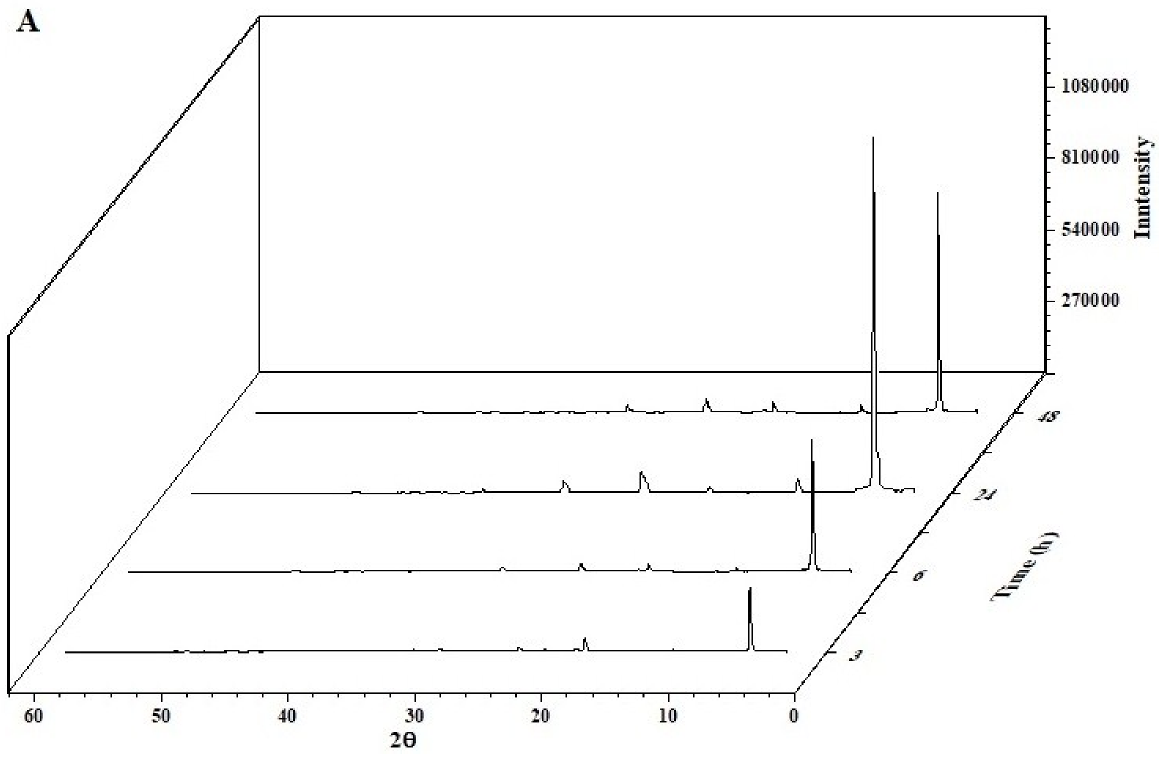

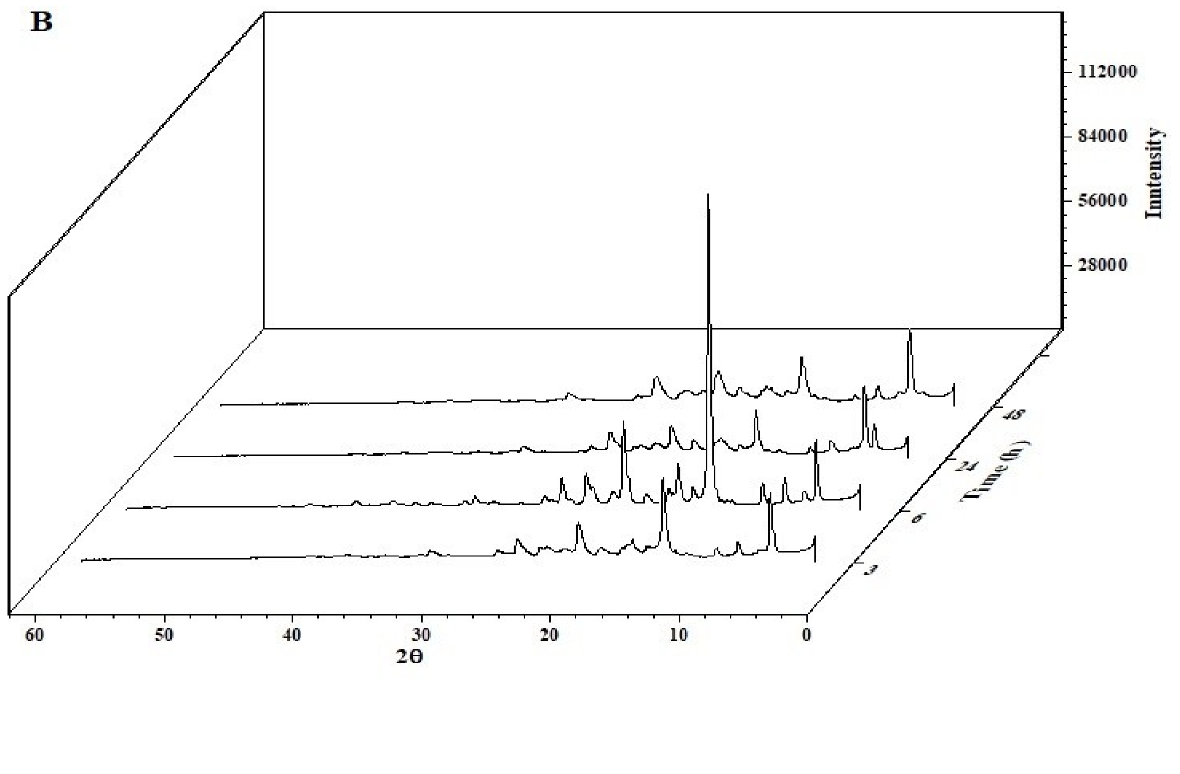

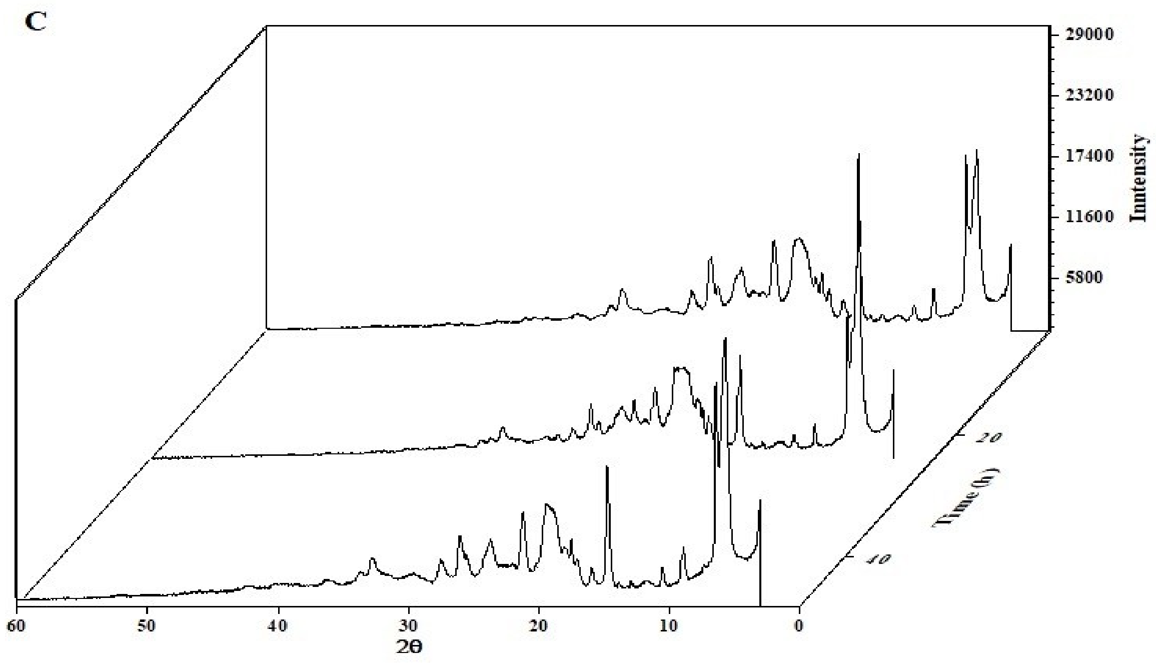
C
29000
23200
17400
11600
5800
Intensity
20
40
Time (h)
60
50
40
30
20
10
0
2θ
